# Steering elementary steps towards efficient alkaline hydrogen evolution via size-dependent Ni/NiO nanoscale heterosurfaces

**DOI:** 10.1093/nsr/nwz145

**Published:** 2019-10-01

**Authors:** Lu Zhao, Yun Zhang, Zhonglong Zhao, Qing-Hua Zhang, Lin-Bo Huang, Lin Gu, Gang Lu, Jin-Song Hu, Li-Jun Wan

**Affiliations:** 1 Beijing National Laboratory for Molecular Sciences (BNLMS), CAS Key Laboratory of Molecular Nanostructure and Nanotechnology, Institute of Chemistry, Chinese Academy of Sciences, Beijing 100190, China; 2 Department of Physics and Astronomy, California State University Northridge, Northridge, CA 91330, USA; 3 Beijing National Research Center for Condensed Matter Physics, Collaborative Innovation Center of Quantum Matter, Institute of Physics, Chinese Academy of Sciences, Beijing 100190, China; 4 College of Chemistry and Materials Science, Sichuan Normal University, Chengdu 610068, China; 5 University of Chinese Academy of Sciences, Beijing 100049, China

**Keywords:** alkaline hydrogen evolution reaction, electrocatalysis, elementary step, Ni/NiO interface

## Abstract

Alkaline hydrogen evolution reaction (HER), consisting of Volmer and Heyrovsky/Tafel steps, requires extra energy for water dissociation, leading to more sluggish kinetics than acidic HER. Despite the advances in electrocatalysts, how to combine active sites to synergistically promote both steps and understand the underlying mechanism remain largely unexplored. Here, Density Functional Theory (DFT) calculations predict that NiO accelerates the Volmer step while metallic Ni facilitates the Heyrovsky/Tafel step. A facile strategy is thus developed to control Ni/NiO heterosurfaces in uniform and well-dispersed Ni-based nanocrystals, targeting both reaction steps synergistically. By systematically modulating the surface composition, we find that steering the elementary steps through tuning the Ni/NiO ratio can significantly enhance alkaline HER activity, and Ni/NiO nanocrystals with a Ni/NiO ratio of 23.7% deliver the best activity, outperforming other state-of-the-art analogues. The results suggest that integrating bicomponent active sites for elementary steps is effective for promoting alkaline HER, but they have to be balanced.

## INTRODUCTION

The electrocatalytic reaction, like other heterogeneous catalytic reactions, consists of multiple elementary reaction steps [1–3]. The reaction kinetics, efficiency and selectivity are dependent on the adsorption/desorption states of reaction intermediates in each elementary step on the catalyst surface [4–6]. One of the ultimate challenges in electrocatalysis is understanding how the physical and chemical states of a catalyst surface affect the adsorption of intermediates, activation energies and energy barriers for each elementary step. Although significant progress has been made on the modulation of extrinsic physical properties and intrinsic electronic structure of electrocatalysts for improving the overall reaction kinetics [7], few reports focus on the specific design and tuning of every elementary step and their influences on catalyst performance [8].

Electrocatalytic alkaline hydrogen evolution reaction (HER) is an ideal model reaction for investigating the relationship between elementary reaction kinetics and catalytic performance because of: (i) the single desired product and its important role in hydrogen-based energy applications [9–13]; (ii) the limited understanding of surface active centers and evaluation/prediction of activity trend on alkaline HER catalysts; (iii) its two to three orders of magnitude lower reaction rate than acidic HER and the controversial reasons [14,15]; and (iv) the relatively clear elementary reactions including the Volmer step (H_2_O + e^−^ → H_ad_ + OH^−^) and Heyrovsky step (H_2_O + H_ad_ + e^−^ → H_2_ + OH^−^) or Tafel step (H_ad_ + H_ad_ → H_2_) [16–18]. Compared with acidic HER, the Volmer step in alkaline electrolyte needs the appropriate adsorption of H_2_O molecules and extra energy to dissociate H_2_O, causing the difficult formation of H_ad_ [14,15,19–22]. Incorporating additional components such as metal oxides/hydroxides or chalcogenides into HER catalysts has been suggested to improve the water dissociation step, and thus catalyst performance [23–28]. For example, Dai's group etc. proposed NiO to assist H_2_O dissociation and desorption of OH_ad_ to enhance alkaline HER activity of Ni-based catalysts [16,29]. However, systematic theoretical and experimental investigation into the inherent reasons underlying the sluggish kinetics of alkaline HER still remain largely unexplored. Whether and how the extra energy barrier for the water dissociation step affects overall HER rate are still inconclusive. The adsorption of OH_ad_ resultant from water decomposition will compete with the adsorption of H_ad_. How to balance the water dissociation and the adsorption of H_ad_/OH_ad_ is still unexplored. If an additional water-dissociation component is included in the catalyst design, whether or not there is an optimal ratio between the components for each elementary step and how to modulate such composition and thus balance the elementary steps for optimizing alkaline HER performance is still undiscovered.

Here, we designed Ni/NiO catalysts with tunable surface chemistry as a model system to obtain insights into these aspects. Density Functional Theory (DFT) calculations are first performed, demonstrating that NiO exhibits a much lower energy barrier for the dissociation of H_2_O into H_ad_/OH_ad_ than Ni while H_ad_ adsorbed on a Ni surface forms H_2_ much more easily than on a NiO surface. Then, we developed a facile strategy to delicately tune the surface composition of Ni/NiO nanocrystals by modulating their sizes. Systematic investigation concludes that the alkaline HER activity of Ni/NiO strongly depends on the ratio of surface Ni and NiO. Surface NiO does enhance HER by facilitating the Volmer step. However, the Volmer step and the Heyrovsky/Tafel step have to be balanced by steering surface Ni and NiO for the best alkaline HER. These results suggest that combining specific active sites for different elementary steps is effective for boosting alkaline HER and their ratio should be balanced for optimal overall reaction. Tuning surface chemistry to steer the elementary steps stands as an option for designing highly efficient alkaline HER electrocatalysts.

## RESULTS AND DISCUSSION

**Figure 1.**
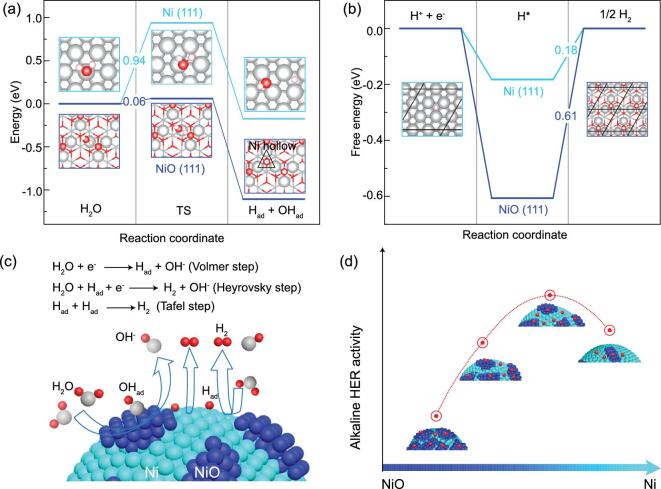
DFT calculations. (A) The energy barrier for breaking the OH-H bond in the Volmer step (water dissociation) and (B) the free energy diagram for hydrogen adsorption (G_H_) on Ni (111) and NiO (111) surfaces in the Heyrovsky step. Gray, red and small white spheres represent Ni, O and H atoms, respectively. (C) Schematic illustration of alkaline HER on Ni/NiO heterosurfaces. (D) Dependence of alkaline HER activity on the surface compositions of Ni/NiO heterosurfaces.

The elementary reaction steps of alkaline HER on the Ni/NiO heterosurface are examined by DFT calculations. The Ni (111) surface and oxygen-terminated octopolar (O-octo) NiO (111) surface [30] are included in the calculations. As shown in Fig. 1a, the energy barrier for breaking the H-OH bond in H_2_O is estimated as 0.94 eV on the Ni (111) surface, rendering H_2_O dissociation into H_ad_ energetically costly. In contrast, the dissociation energy barrier of H_2_O on the NiO (111) surface is only 0.06 eV, suggesting that the NiO surface can drastically enhance water dissociation into H_ad_. As depicted in the insets of Fig. 1a, DFT calculations indicate that at the initial state, H_2_O is adsorbed on the Ni site of the NiO (111) surface. And at the final state, the dissociated H_ad_ and OH_ad_ occupy the exposed O site and the 3-fold hollow Ni site, respectively. Since OH_ad_ prefers to bind to the O vacancy on the O-octo surface, the adsorption energy of the final state is lowered, resulting in the reduced energy barrier, following the Brønsted−Evans−Polanyi relationship [31–33]. Subsequently, the produced H_ad_ could form H_2_ through either the Heyrovsky or the Tafel step. In both steps, the free energy of hydrogen adsorption, G_H_, has been identified as the activity descriptor [34,35]. Fig. 1b shows the calculated G_H_ on Ni (111) and NiO (111) surfaces. It is found that G_H_ on the NiO surface is much more negative than the optimal value (G_H_ = 0), suggesting that H_2_ generation on the NiO surface would be difficult. In contrast, G_H_ on Ni (111) is much less negative and closer to the optimal value. Thus, the combination of H_ad_ to form H_2_ is much easier on the Ni (111) surface, compared to the NiO surface. Therefore, if one can design Ni/NiO heterostructured surfaces which synergistically promote both reaction steps, the overall alkaline HER could potentially be enhanced. As illustrated in Fig. 1c, this can be achieved by steering the Volmer step (the dissociation of H_2_O) on the NiO surfaces while the evolved H_ad_ could form H_2_ on adjacent Ni surfaces via the Heyrovsky or Tafel step. The challenge is to tune such Ni/NiO heterosurfaces so that an optimal balance of Ni and NiO components can be achieved to attain the best activity (Fig. 1d).

**Figure 2.**
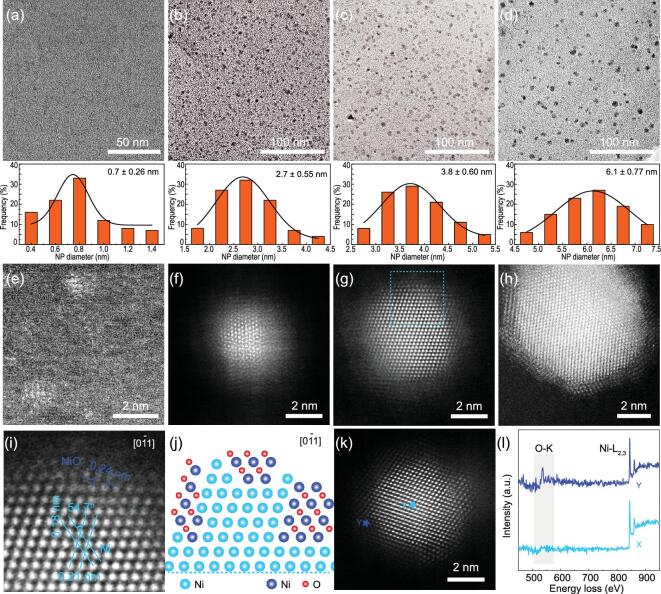
Structural characterizations. (a–d) Typical TEM images of Ni/NiO nanocrystals in different sizes: (A) 0.7, (B) 2.7, (C) 3.8 and (D) 6.1 nm. Below: corresponding histograms of size distribution. (e–h) Typical HAADF-STEM images of Ni/NiO nanocrystals shown in (a–d), respectively. (I) Zoom-in HAADF-STEM image as marked by the square in (G). (J) Schematic illustration of heterostructure of Ni/NiO. (K) Typical HAADF-STEM image for EELS analysis and the corresponding Ni-L and O-K EELS spectra (L) of Ni/NiO-3.8. Spectra were collected on the point X and Y as marked in (K).

We developed here a facile strategy to achieve well-dispersed Ni/NiO nanocrystals and discovered that the ratio of surface Ni and NiO can be delicately modulated by size control and native oxidation. Four types of Ni/NiO nanocrystals in different sizes were prepared by loading a Ni source on a selected nanoporous carbon substrate and thermal annealing in H_2_/Ar flow at controlled conditions to obtain metallic Ni nanocrystals, followed by native oxidation to form a Ni/NiO heterosurface upon exposure to air (see Methods for details and Supplementary Figs 1 and 2 in the online supplementary material) [36,37]. The obtained samples were denoted as Ni/NiO-x, where x represents the average size of nanocrystals in a nanometer. In order to exclude the effect of Ni content on catalytic performances of the catalysts, four Ni/NiO samples were prepared with the same amount of Ni source. The similar Ni contents in Ni/NiO-0.7, −2.7, −3.8 and −6.1 samples are further confirmed as 10.51, 11.96, 11.08 and 12.35 wt%, respectively, by thermal gravimetric (TG) analysis (seen online supplementary material for details, Supplementary Fig. 3). As shown in transmission electron microscopy (TEM) images and size-distribution histograms (Fig. 2a–d and Supplementary Figs 4–8), nanocrystals in all four samples are uniformly and highly dispersed on the substrate without aggregation. The size distribution analyses over 400 nanocrystals gave average sizes of 0.7, 2.7, 3.8 and 6.1 nm, respectively. Detailed atomic-scale structural features of these nanocrystals were investigated by the high-angle annular dark-field scanning transmission electron microscopy (HAADF-STEM) technique. Except for sample Ni/NiO-0.7 (Fig. 2e and Supplementary Fig. 5), a clear contrast difference between the inner and outer part is observed for the other three samples (Fig. 2f–h), suggesting a core/shell-like structure. The core parts are well crystallized in terms of the atomic arrangement in good order and can be assigned to metallic Ni. Massive defects with lower contrast are observed in the shell parts, indicating their discontinuous and disordered nature. Furthermore, it is found that the thickness of such shells depends on the size of the nanocrystals. It is known that the surface free energy of a metal nanoparticle increases dramatically with the decrease of particle size, causing it to be more active for chemical reactions, such as oxidation [38]. The smaller nanocrystal is more easily oxidized during native oxidation, giving the thicker oxide shell. Taking Ni/NiO-3.8 as an example, a typical zoom-in HAADF-STEM image (Fig. 2i) shows interplanar distances of 0.21 and 0.18 nm in a characteristic angle of 54.7°, which can be assigned well to the (111) and (200) planes of face-centered-cubic (fcc) Ni (JCPDS No. 04–0850). The lattice fringes with a spacing of 0.24 nm at the porous shell part can be attributed to the (111) planes of fcc NiO (JCPDS No. 47–1049). These observations suggest the formation of a Ni/NiO heterostructure. During the native oxidation, oxygen could adsorb first on the surface of Ni nanocrystals and diffuse into the sub-layer region to generate NiO layers, epitaxially growing on the Ni surface as depicted in Fig. 2j [37,39–42]. The electron energy-loss spectroscopy (EELS) is further performed to investigate such heterostructure. As shown in Fig. 2k and l, the signals of Ni-L2,3 and O-K edges (at 856 and 532 eV, respectively) are collected at both spot X on core and spot Y on shell while the intensity of the O-K edge at spot Y is much stronger than that at spot X, corroborating the core/shell structure of the Ni/NiO nanocrystal.

**Figure 3.**
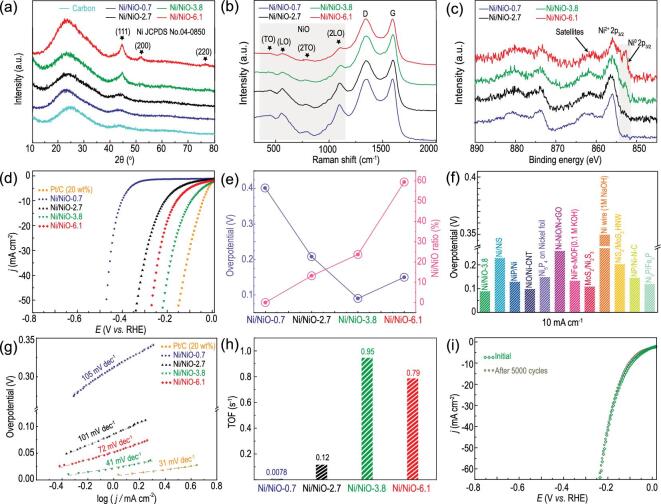
Spectroscopic characterizations and electrocatalytic performance for HER. (A) XRD patterns, (B) Raman spectra, and (C) high-resolution Ni 2p XPS spectra of Ni/NiO nanocrystals. (D) Polarization curves and (E) the relationship between overpotentials at 10 mA cm^−2^ and Ni/NiO ratio of Ni/NiO-0.7, −2.7, −3.8 and −6.1. (F) Overpotential comparison of Ni/NiO-3.8 with state-of-the-art Ni-based HER catalysts. (G) Tafel plots and (H) turn-over frequency (TOF) (at 200 mV overpotential) of different Ni/NiO nanocrystals. (I) Polarization curves before and after 5000 CV cycles of Ni/NiO-3.8. The benchmark Pt/C catalyst was also evaluated in (d and g) for comparison.

The X-ray diffraction (XRD) patterns of four samples are shown in Fig. 3a. Except for diffraction peaks at 26.1 and 44.7° from the carbon substrate, three broadened peaks at 44.5, 51.8 and 76.4° are distinguished for sample Ni/NiO-3.8 and Ni/NiO-6.1, corresponding to the diffractions of (111), (200) and (220) planes of fcc Ni (JCPDS No. 04–0850). The intensity decreases as nanocrystal sizes decrease. No clear XRD peaks are observed for sample Ni/NiO-0.7 and Ni/NiO-2.7 probably due to the small size. No NiO signals are collected for all samples, consistent with the discontinuous and short-range order feature of the NiO shell as observed by HAADF-STEM. Raman spectra are subsequently recorded to identify NiO (Fig. 3b). Besides two peaks at 1350 and 1580 cm^−1^ from the carbon substrate [43,44], four characteristic Raman peaks appeared at 440, 565, 770 and 1108 cm^−1^ for all samples, which correspond well to the first-order transverse optical (TO), longitudinal optical (LO), 2TO, and 2LO phonon vibrations of NiO, respectively [45,46]. The relative intensities of these phonon modes increase as nanocrystal sizes decrease, suggesting the increase of NiO content in order [47]. The chemical state of element Ni and their relative contents are investigated by X-ray photoelectron spectroscopy (XPS). As shown in Fig. 3c, Ni^0^ signals at 852.9 and 870.9 eV and Ni^2+^ signals at 856.2 and 874.6 eV are clearly identified in high-resolution Ni 2p XPS spectra, except for Ni/NiO-0.7 where no Ni^0^ signal is discerned. The signal from the O-Ni bond in a deconvoluted high-resolution O 1 s XPS spectrum suggests that the detected Ni^2+^ should come from NiO (Supplementary Fig. 9). As expected, the signal intensity for Ni^2+^ enhances with the decrease of nanocrystal size, which means the increase of NiO content in turn. This matches well with STEM, XRD, and Raman analyses. The fittings of high-resolution Ni 2p spectra (Supplementary Fig. 10) give the surface Ni/NiO ratios of ∼0, 13.2, 23.7 and 59.5% for samples Ni/NiO-0.7, −2.7, −3.8 and −6.1, respectively (Supplementary Table 1 in the online supplementary material). The above results indicate that four Ni/NiO nanocrystal samples with tunable surface Ni/NiO ratios have been successfully prepared, which will be subsequently used as model catalysts for investigating the structure–activity relationship between Ni/NiO-modulated elementary steps and alkaline HER activity.

The alkaline electrocatalytic HER activities of Ni/NiO samples and the state-of-the-art Pt/C catalyst were examined in 1 M KOH. The electrochemically active surface areas (ECSAs) were determined by measuring the double-layer capacitance (C_dl_) in the non-faradaic region since it is linearly proportional to ECSA. The C_dl_ is 111, 115, 127 and 124 mF cm^−2^ for Ni/NiO-0.7, −2.7, −3.8 and −6.1, respectively (Supplementary Figs 11 and 12), suggesting that these samples share similar ECSAs and excluding the influence of ECSA on the activity. The polarization curves are shown in Fig. 3d and the performance parameters are summarized in Fig. 3e. Clearly, Ni/NiO-0.7 with no detectable Ni^0^ component displays the worst HER activity in terms of the largest onset potential of −305 mV and overpotential of 401 mV at 10 mA cm^−2^ (all potentials are versus to reversible hydrogen electrode (RHE) in context), indicating a metallic Ni component for accelerating the Tafel (or Heyrovsky) step may be necessary for enhancing alkaline HER as indicated by DFT calculations. As the Ni^0^ content increases with the increase of nanocrystal size, the HER activity firstly increases, then degrades. Ni/NiO-3.8 with a Ni/NiO ratio of 23.7% exhibits the best HER catalytic activity in terms of the mostly positive onset potential (−11 mV) and the lowest overpotential of 90 mV at 10 mA cm^−2^. This performance is comparable to the benchmark Pt/C catalyst and outperforms recently reported Ni-based catalysts (Fig. 3f and Supplementary Table 2). Mass-normalized activity is also calculated for ruling out the influence of slight variations in the mass of Ni/NiO samples (Supplementary Fig. 13). Similarly, Ni/NiO-3.8 displays the best HER activity with an overpotential of 34 mV at 50 mA mg^−1^. Further increasing Ni/NiO ratio to 59.5% in Ni/NiO-6.1, HER activity appreciably decreases, implying that Ni and the NiO component should be balanced for alkaline HER. This degradation could be attributed to the insufficient NiO active component for the Volmer step. As shown in the Tafel plots (Fig. 3g), the reaction kinetics for these samples is in line with their catalytic activity, corroborating that the alkaline HER activity on Ni/NiO is dependent on its surface chemistry. Moreover, the values of the Tafel slopes suggest that the HER process follows a Volmer–Heyrovsky mechanism [48–51]. Electrochemical impedance spectroscopy (EIS) of Ni/NiO samples were measured to further evaluate the reaction kinetics. Compared with the sample Ni/NiO-2.7 and −6.1, Ni/NiO-3.8 exhibits the smallest charge transfer resistances (Rct), indicating its fastest charge transfer kinetics (Supplementary Fig. 14). In order to exclude the influence of active site number on HER activity trend, the turnover frequency (TOF) is further calculated at the overpotential of 200 mV for four samples and presented in Fig. 3h (see the online supplementary material for details). Consistently, Ni/NiO-3.8 exhibits the highest TOF value of 0.95 s^−1^ and Ni/NiO-0.7 shows the lowest TOF value of 0.0078 s^−1^, confirming Ni/NiO heterosurface-dependent alkaline HER activity. The stability test was subsequently performed on the best catalyst Ni/NiO-3.8. It shows a negligible degradation after standard 5000 cyclic voltammetry (CV) cycles (Fig. 3i). TEM images prove that the morphology and size of Ni/NiO nanocrystals show no appreciable change after stability test (Supplementary Fig. 15). These results indicate the excellent durability of the Ni/NiO catalyst for alkaline HER.

**Figure 4.**
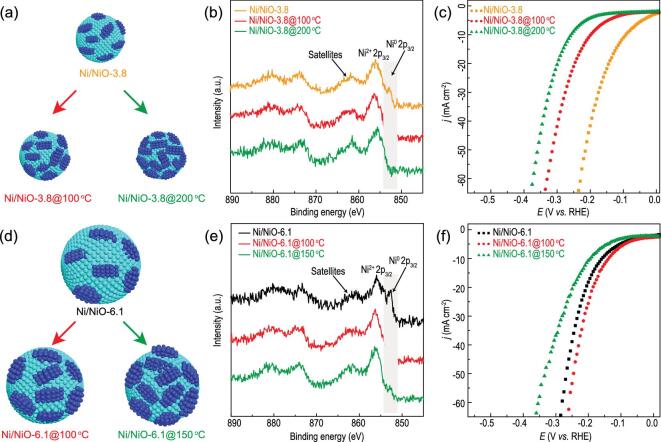
Modulation of surface chemistry and the influence on HER activity. Schematic illustrations of the surface modulations on (A) Ni/NiO-3.8 and (D) −6.1 by controlled post-oxidization, respectively. (B and E) High-resolution Ni 2p XPS spectra and (C and F) polarization curves of Ni/NiO-3.8 and −6.1 with different oxidation degrees, where the suffix after the sample name represents the oxidation temperature.

To further investigate the dependence of alkaline HER activity on the ratio of Ni and NiO and rule out the uncertainties in comparison, we used the same catalyst to achieve the different Ni/NiO ratios by delicate oxidation at controlled conditions (see Methods for details). For catalyst Ni/NiO-3.8, the Ni/NiO ratio decreases from 23.7 to 11.5% and ∼0 (calculated from XPS data) after oxidation at 100 and 200°C, respectively (Fig. 4a and b, Supplementary Figs 16 and 17 and Table 1). Raman spectra corroborate the increase of NiO content as oxidation temperature increases (Supplementary Fig. 18). The electrochemical measurement clearly indicates that HER activity decreases as the Ni/NiO ratio decreases (Fig. 4c), consistent with the cases in Ni/NiO-2.7 and Ni/NiO-0.7. Moreover, for catalyst Ni/NiO-6.1, the Ni/NiO ratio decreases from 59.5% to 27.8% and 15.8% after oxidation at 100 and 150°C, respectively (Fig. 4d and e and Supplementary Figs 19 and 20). As also confirmed by Raman spectra (Supplementary Fig. 21), NiO content increases as oxidation temperature increases. However, HER activity firstly improves when the Ni/NiO ratio reaches ∼20% and then significantly degrades when it further reduces (Fig. 4f). Moreover, it is worthy to note that the Ni/NiO-6.1@100°C has a slightly higher Ni/NiO ratio than Ni/NiO-3.8 (27.8% vs. 23.7%) while its HER activity (138 mV at 10 mA cm^−2^) and TOF (deducting the effect of active site number on HER activity) (0.92 s^−1^) are slightly lower than Ni/NiO-3.8 (90 mV at 10 mA cm^−2^, 0.95 s^−1^). Compared with Ni/NiO-3.8, the HER activity of Ni/NiO-6.1@ 150°C with a Ni/NiO ratio of 15.8% is drastically degraded. These results indicate that the best surface Ni/NiO ratio for alkaline HER on Ni/NiO nanocrystals should be close to that in Ni/NiO-3.8 (23.7%). Too much NiO component will cause insufficient Ni active sites for the Heyrovsky step. The excessive OH_ad_ from water decomposition on NiO also limits the adsorption of H_ad_, causing ineffective H_2_ formation via the Heyrovsky step. On the other hand, if the Ni component is excessive, water dissociation (Volmer step) becomes a rate-limiting step, causing insufficient rate of H_ad_ formation. Therefore, the two components responsible for different elementary steps in alkaline HER should be balanced to achieve the highest HER activity and the best Ni/NiO ratio in our model catalysts is ∼20%.

## CONCLUSION

In summary, we systematically investigated the influence of the modulation of elementary steps on alkaline HER activity using controlled Ni/NiO bi-component model catalysts. DFT calculations suggested that the NiO component could accelerate the water dissociation step while the metallic Ni component could facilitate H_2_ formation from the adsorbed H_ad_. A series of well-dispersed and uniform Ni/NiO nanocrystals with different surface Ni/NiO ratios were successfully prepared as model catalysts by size controlling and native oxidation. Systematic electrochemical evaluations discovered that the alkaline HER activity closely depended on the composition of such Ni/NiO heterosurfaces, where the two components should be balanced for achieving the optimal performance. This finding was further corroborated by delicately steering the surface chemistry of the same catalyst via post-oxidation so as to rule out the possible uncertain effects from the catalyst variations. The detailed comparison showed that Ni/NiO nanocrystals with an average size of 3.8 nm exhibited superior alkaline HER activity and stability, and the optimal Ni/NiO ratio for alkaline HER on model catalysts was ∼20%. These results suggested that steering the elementary steps by tuning the surface chemistry of catalysts is effective for advancing their alkaline HER performance, shedding light on the design of earth-abundant efficient HER electrocatalysts and the understanding of the structure–activity relationships therein.

## METHODS

### Materials

Potassium citrate (K_3_C_6_H_5_O_7_·H_2_O), nickel (II) nitrate hexahydrate (Ni(NO_3_)_2_·6H_2_O), Nafion solution (5 wt%) and potassium hydroxide (KOH) were purchased from Alfa Aesar. Concentrated sulfuric acid (H_2_SO_4_) was obtained from Sigma-Aldrich. Commercial 20 wt% Pt/C was purchased from Johnson Matthey Company. The deionized water (18.2 MΩ) was produced by the Millipore Milli-Q water purification system. All reagents were used directly without further purification.

### Synthesis of nanoporous carbon support

Nanoporous carbon substrate was prepared by pyrolyzing 8 mmol of K_3_C_6_H_5_O_7_·H_2_O at 800°C for 1 h in a tube furnace under Ar atmosphere [36]. The black solid product was washed with H_2_SO_4_ solution (0.5 M) and water several times to remove inorganic impurities. The pyrolysis of potassium citrate at 800°C under Ar leads to honeycomb-like carbon with some inorganic impurities such as potassium compounds. These impurities can be completely removed by washing with 0.5 M H_2_SO_4_. After drying at 60°C, the carbon support with micropores and nanopores was achieved.

### Synthesis of Ni/NiO samples

The Ni/NiO samples were in situ prepared by adsorbing Ni(NO_3_)_2_ on nanoporous carbon support as a metal source and thermal annealing under an H_2_/Ar atmosphere. Typically, 60 mg of nanoporous carbon was dispersed in 5 mL of water containing 0.85 mmol Ni(NO_3_)_2_·6H_2_O, then sonicated for 30 min to get a homogeneous black suspension. The suspension was then centrifuged and dried at 60°C. The obtained powder was placed into a tube furnace and heated to the set temperature (200, 250, 300, 400°C) in a flow of 10% H_2_/Ar flow (100 sccm). After 2 h of thermal annealing, the product was cooled down to room temperature and exposed to the atmosphere. The corresponding products prepared at various annealing temperatures were denoted as Ni/NiO-0.7, −2.7, −3.8 and −6.1 according to their average size, respectively.

### Preparation of control samples by *ex situ* oxidation

In order to unambiguously investigate the influence of the surface Ni/NiO ratio on alkaline HER and preclude the differences in the number and size of nanocrystals in different samples during comparison, the surface Ni/NiO ratio was further modulated by *ex situ* oxidization of the same sample. Both samples Ni/NiO-3.8 and −6.1 were *ex situ* oxidized at varied temperatures to prepare the corresponding control samples. Typically, 60 mg of Ni/NiO-3.8 was placed into a porcelain combustion boat and heated to the set temperature (100 and 200°C) at a ramping rate of 1°C min^−1^ in a muffle furnace. After 6 h and 10 h oxidation, respectively, the oxidized product was achieved. The samples were denoted as Ni/NiO-3.8@100°C and −200°C according to the oxidation temperature, respectively. Another two control samples Ni/NiO-6.1@100°C and −150°C were obtained via the same procedure except for using Ni/NiO-6.1 instead of Ni/NiO-3.8 and annealing at 100 or 150°C for 10 h, respectively.

### Characterizations

Powder XRD experiments were performed on a Regaku D/Max-2500 diffractometer equipped with a Cu Kα1 radiation (λ = 1.54056 Å) (Rigaku, Japan). The morphologies were characterized on a SEM (S4800, JEOL, Japan) operated at 10 kV and a TEM (JEM-2100F, JEOL, Japan) working at an accelerating voltage of 200 kV. HAADF-STEM and EELS spectra were collected on a JEOL ARM200F electron microscope (JEOL, Japan) operated at 200 kV with a cold field emission gun and double hexapole Cs correctors (CEOS GmbH, Germany). The attainable spatial resolution defined by the probe-forming objective lens is better than 80 pm. XPS spectra were recorded on an ESCALab220i-XL electron spectrometer (VG Scientific, UK) using an Al Kα radiation. Raman spectra were obtained on a Lab-RAM HR Evolution spectrometer (HORIBA, France) with a laser excitation wavelength of 532 nm. The Ni/NiO loadings were measured via TG analysis on a Netzsch DSC214 instrument (NETZSCH, Germany) under air flow from 40 to 800°C at a ramp rate of 10°C min^−1^.

### Electrochemical measurements

All electrochemical measurements were recorded at room temperature on a rotating ring-disk electrode rotator (RRDE-3A, ALS, Japan) by a standard three-electrode cell system connected to an electrochemical workstation (Autolab PGSTAT302N, Metrohm, Netherlands). A rotating disk electrode (RDE) (4 mm in diameter) with catalysts acted as the working electrode. Hg/HgO (1 M NaOH) electrode and graphite electrode were used as reference and counter electrodes, respectively. The working electrodes were prepared as follows: catalyst ink was prepared by mixing 2 mg catalysts and 800 μL ethanol in a glass vial and sonicating for 30 min. The 40 μL ink and 2 μL (0.5 wt% of Nafion solution) was dropped on freshly polished RDE and dried for 10 min in the air to obtain catalyst loading of 800 μg cm^−2^. For comparison, commercial Johnson-Matthey Pt/C (20 wt%) was also measured with a loading of 25.5 μg_pt_ cm^−2^. The HER performance was evaluated by linear sweep voltammetric scanning from 0.25 to −0.6 V at a scan rate of 5 mV s^−1^ at 1600 rpm in 1 M KOH. Cyclic voltammetric measurements were conducted at various scan rates (4, 6, 8, 10, 12, 14 and 16 mV s^−1^) in a potential window (−0.02 to 0.01 V) for calculating the C_dl_. CV was also used to evaluate the catalyst stability. EIS was performed at an overpotential of 0.226 V in the frequency range of 0.1 to 10^5^ Hz. All the polarization curves were obtained with the ohmic potential drop (ir) correction. All potentials were quoted with respect to a reversible hydrogen electrode.

### Computational methods

Spin-polarized DFT calculations were performed using the Vienna ab initio Simulation Package (VASP) [52] with the projector-augmented wave pseudopotentials [53]. The exchange-correlation interaction was described by the Perdew–Burke–Ernzerhof (PBE) functional [54]. For NiO, the PBE functional together with the Hubbard-U method [55] was employed to minimize the self-interaction error. An effective Hubbard-U parameter, U_eff_ = 6.45 eV, was applied for the d-electrons of Ni atoms, which is same as previous studies [30,56]. A plane-wave energy cutoff of 400 eV was used for all calculations. The Ni (111) and NiO (111) surfaces were modeled by four-atomic-layer and five-atomic-layer slabs, respectively. The adjacent computational slabs were separated by a 15 Å vacuum in the normal direction of the surface. The atoms in the top three layers were fully relaxed, while the rest of the atoms were fixed in the equilibrium positions. The Brillouin-zone was sampled with a 3 × 3 × 1 k-mesh according to the Monkhorst–Pack scheme [57].

The transition state for water dissociation was determined using the Climbing-Image-Nudged Elastic Band (CI-NEB) method [58]. The transition states were searched with six images. The hydrogen adsorption free energy G_H_ was calculated as

$$\begin{eqnarray*}
{G_H} &=& {\rm{\ }}E\left[ {{\rm{surf}} + H} \right] - E\left[ {{\rm{surf}}} \right] - E\left[ {{H_2}} \right]/2 \nonumber\\
&& +\, {\textit{ZPE}} + \mathop \smallint \nolimits {C_P}dT - TS,
\end{eqnarray*}$$

where *E*[surf + H] and *E*[surf] are the total energies of the surface with and without the H adsorbate, respectively. *E*[H_2_] is the total energy of a hydrogen molecule. ZPE, C_P_ and −*TS* are the zero-point energy, heat capacity and entropy corrections calculated based on the molecular vibration analysis at *T* = 300 K [59]. The free energy diagram for HER was obtained by using the computational hydrogen electrode (CHE) model. In the CHE model, the chemical potential of a proton–electron pair is defined in equilibrium with half of that of gaseous H_2_ at 101 325 Pa and any pH values. The chemical potential is shifted by −*eU* (*e* is the elementary positive charge) when an external potential *U* is applied.

## Supplementary Material

nwz145_Supplemental_File
