# Supplementary material for: Steering elementary steps towards efficient alkaline hydrogen evolution via size-dependent Ni/NiO nanoscale heterosurfaces
Source: Natl Sci Rev. 2019 Oct 1;7(1):27–36. doi: 10.1093/nsr/nwz145 (PMC8288842; doi:10.1093/nsr/nwz145)
Supplement: nwz145_Supplemental_File [file nwz145_supplemental_file.docx]

**Supporting Information**

**Steering Elementary Steps towards Efficient Alkaline Hydrogen Evolution via Size-Dependent Ni/NiO Nanoscale Heterosurfaces**

Lu Zhao,^1,5^ Yun Zhang,^1,4^ Zhonglong Zhao,^2^ Qing-Hua Zhang,^3^ Lin-Bo Huang,^1,5^ Lin Gu,^3^ Gang Lu,^2^ Jin-Song Hu,^1,5,*^ Li-Jun Wan^1,5,*^

^1^ Beijing National Laboratory for Molecular Sciences (BNLMS), CAS Key Laboratory of Molecular Nanostructure and Nanotechnology, Institute of Chemistry, Chinese Academy of Sciences, Beijing 100190, China.

^2^ Department of Physics and Astronomy, California State University Northridge, Northridge, California 91330, USA.

^3^ Beijing National Research Center for Condensed Matter Physics, Collaborative Innovation Center of Quantum Matter, Institute of Physics, Chinese Academy of Sciences, Beijing 100190, China.

^4^ College of Chemistry and Materials Science, Sichuan Normal University, Chengdu 610068, China.

^5^ University of Chinese Academy of Sciences, Beijing 100049, China.

^*^Corresponding author. E-mail: hujs@iccas.ac.cn; wanlijun@iccas.ac.cn

**TOF calculation [1]**

The per-site TOFs were calculated using the following formula:

$$TOF=\frac{\#number of total hydrogen turnover per\mathrm{cm}^{2}}{\#number of active sites per\mathrm{cm}^{2}}=\frac{\#H_{2}\times\left| j \right|}{active sites}$$

The number of total hydrogen turnovers ($\#H_{2}$) was calculated from the current density according to the formula:

$$\#H_{2}=\left( j\frac{\mathrm{mA}}{\mathrm{cm}^{2}} \right)\left( \frac{1C/s}{1000 mA} \right)\left( \frac{{1 mol e}^{-}}{96485.3 C} \right)\left( \frac{1 mol H_{2}}{2 mol e^{-}} \right)\left( \frac{6.02\times{10}^{23}\mathrm{molecules}H_{2}}{1 mol H_{2}} \right)$$

$$=3.12\times{10}^{15}\frac{H_{2}/s}{\mathrm{cm}^{2}}\mathrm{per}\frac{\mathrm{mA}}{\mathrm{cm}^{2}}$$

The surface atomic percentages were determined based on the [geometrical](javascript:;) [configuration](javascript:;)s for different nanocrystal sizes as follows:

| Samples | | Ni/NiO-0.7 | Ni/NiO-2.7 | Ni/NiO-3.8 | Ni/NiO-6.1 |
| --- | --- | --- | --- | --- | --- |
| Truncated octahedron-nanocrystals | 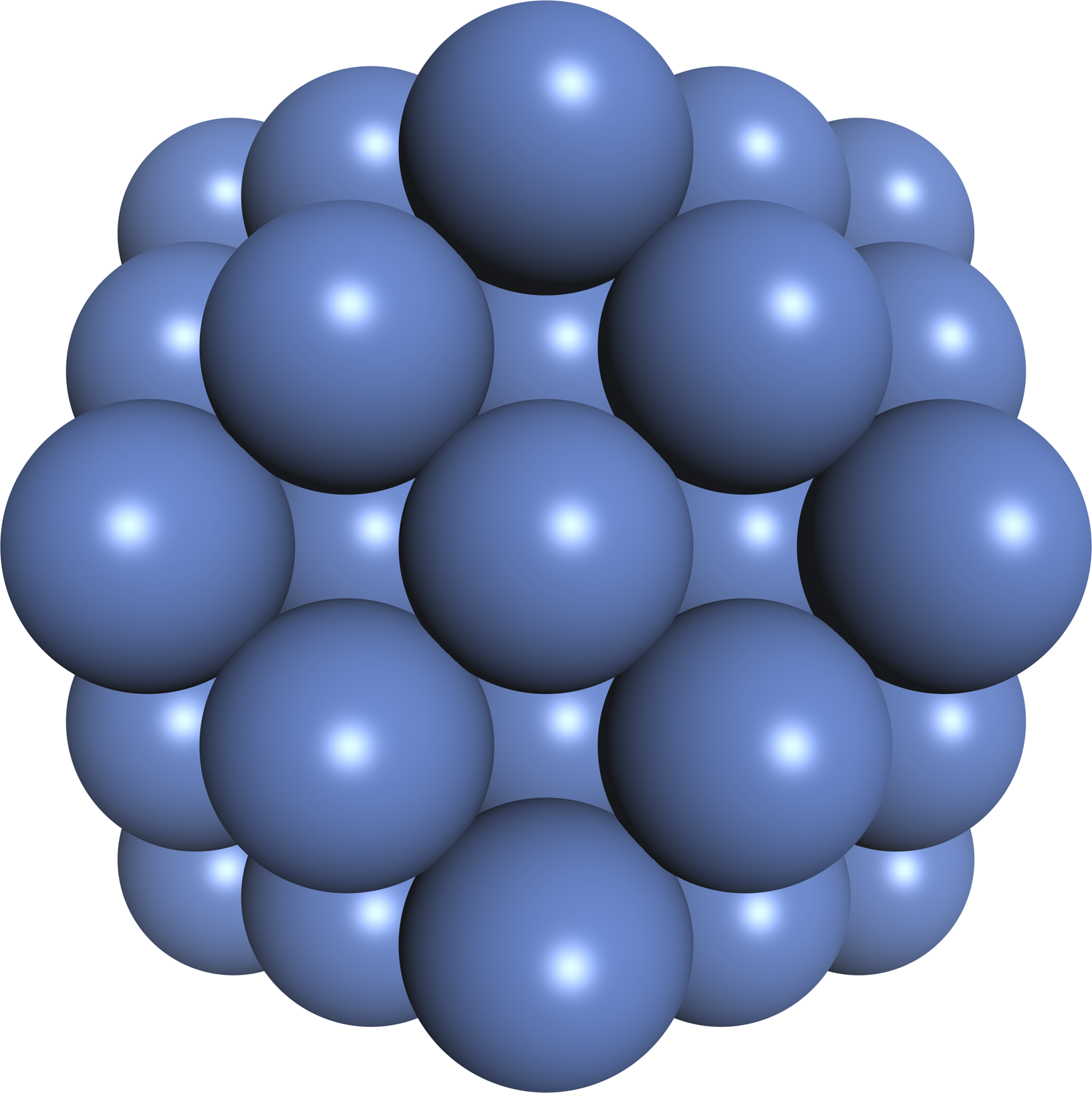 | | 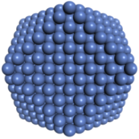 | 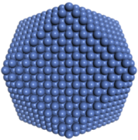 | 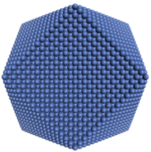 |
| Number of atoms | 55 | | 923 | 2869 | 12431 |
| Number of surface atoms | 36 | | 213 | 426 | 1077 |

Ni contents were calculated from thermogravimetry analyses:

| Samples | Ni/NiO-0.7 | Ni/NiO-2.7 | Ni/NiO-3.8 | Ni/NiO-6.1 |
| --- | --- | --- | --- | --- |
| m % (Ni) | 10.51 wt% | 11.96 wt% | 11.07 wt% | 12.35 wt% |

The number of Ni atoms was calculated from the deposited mass and molar mass:

$$\#Ni/NiO-x=\left( \frac{8\times{10}^{-4}\frac{g}{\mathrm{cm}^{2}}\times m \% (Ni)}{58.69 \frac{g}{\mathrm{mol}}} \right)\left( 6.02\times{10}^{23}\frac{Ni atom}{\mathrm{mol}} \right)$$

$$=8.2\times{10}^{18}\times m \% (Ni) Ni atoms per \mathrm{cm}^{2}$$

Ni/NiO-0.7: $8.6\times{10}^{17} Ni atoms per \mathrm{cm}^{2}$;

Ni/NiO-2.7: $9.8\times{10}^{17} Ni atoms per \mathrm{cm}^{2}$;

Ni/NiO-3.8: $9.1\times{10}^{17} Ni atoms per \mathrm{cm}^{2}$;

Ni/NiO-6.1: $1.0\times{10}^{18} Ni atoms per \mathrm{cm}^{2}$.

The number of active sites:

Ni/NiO-0.7:

$$8.6\times{10}^{17} Ni atoms per \mathrm{cm}^{2}\times\frac{36}{55}=5.63 \times{10}^{17} active sites per \mathrm{cm}^{2}$$

Ni/NiO-2.7:

$$9.8\times{10}^{17} Ni atoms per \mathrm{cm}^{2}\times\frac{213}{923}=2.26 \times{10}^{17} active sites per \mathrm{cm}^{2}$$

Ni/NiO-3.8:

$$9.1\times{10}^{17} Ni atoms per \mathrm{cm}^{2}\times\frac{429}{2869}=1.36 \times{10}^{17} active sites per \mathrm{cm}^{2}$$

Ni/NiO-6.1:

$$1.0\times{10}^{18} Ni atoms per \mathrm{cm}^{2}\times\frac{1077}{12431}=8.70 \times{10}^{16} active sites per \mathrm{cm}^{2}$$

At the overpotential of 200 mV, the HER current density for Ni/NiO-0.7 is 1.4 mA cm^-2^. The TOF was calculated to be:

$$TOF=\frac{3.12\times{10}^{15}\frac{H_{2}/s}{\mathrm{cm}^{2}}\mathrm{per}\frac{\mathrm{mA}}{\mathrm{cm}^{2}}\times1.4\frac{\mathrm{mA}}{\mathrm{cm}^{2}}}{5.63 \times{10}^{17}\mathrm{sites} \mathrm{cm}^{-2}}=0.0078s^{-1}$$

Similarly, the HER current density for Ni/NiO-2.7, -3.8, -6.1, and Ni/NiO-6.1@100 ^o^C are 9.0, 41.5, 22.1, and 25.6 mA cm^-2^ at the overpotential of 200 mV, respectively. The TOFs were calculated to be:

Ni/NiO-2.7:

$$TOF=\frac{3.12\times{10}^{15}\frac{H_{2}/s}{\mathrm{cm}^{2}}\mathrm{per}\frac{\mathrm{mA}}{\mathrm{cm}^{2}}\times9.0\frac{\mathrm{mA}}{\mathrm{cm}^{2}}}{2.26 \times{10}^{17}\mathrm{sites} \mathrm{cm}^{-2}}=0.12s^{-1}$$

Ni/NiO-3.8:

$$TOF=\frac{3.12\times{10}^{15}\frac{H_{2}/s}{\mathrm{cm}^{2}}\mathrm{per}\frac{\mathrm{mA}}{\mathrm{cm}^{2}}\times41.5\frac{\mathrm{mA}}{\mathrm{cm}^{2}}}{1.36\times{10}^{17}\mathrm{sites} \mathrm{cm}^{-2}}=0.95s^{-1}$$

Ni/NiO-6.1:

$$TOF=\frac{3.12\times{10}^{15}\frac{H_{2}/s}{\mathrm{cm}^{2}}\mathrm{per}\frac{\mathrm{mA}}{\mathrm{cm}^{2}}\times22.1 \frac{\mathrm{mA}}{\mathrm{cm}^{2}}}{8.70\times{10}^{16}\mathrm{sites} \mathrm{cm}^{-2}}=0.79s^{-1}$$

Ni/NiO-6.1@100 ^o^C:

$$TOF=\frac{3.12\times{10}^{15}\frac{H_{2}/s}{\mathrm{cm}^{2}}\mathrm{per}\frac{\mathrm{mA}}{\mathrm{cm}^{2}}\times25.6\frac{\mathrm{mA}}{\mathrm{cm}^{2}}}{8.70\times{10}^{16}\mathrm{sites} \mathrm{cm}^{-2}}=0.92s^{-1}$$

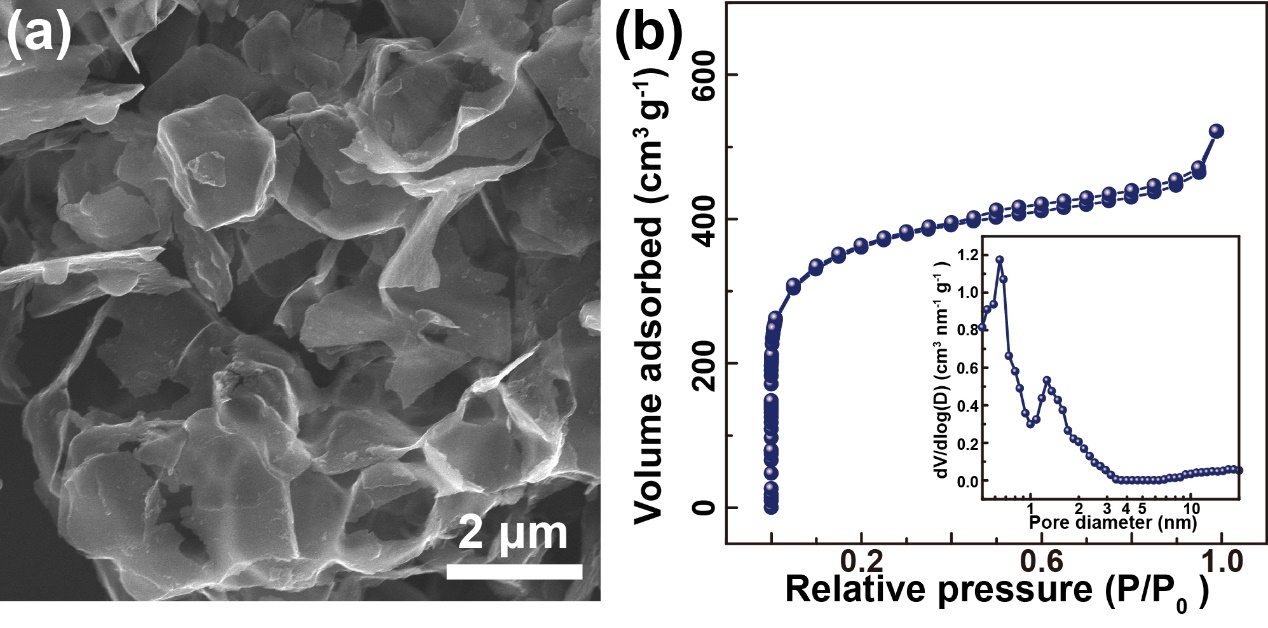


**Supplementary Figure 1.** (a) SEM image and (b) N_2_ adsorption–desorption isotherm (inset: pore size distribution curve) of nanoporous carbon substrate. The high surface areas (1310 m^2^ g^−1^) and pore volume (0.58 cm^3^ g^−1^) have been obtained for nanoporous carbon substrate with abundant nanopores centered at 0.6 and 1.3 nm.


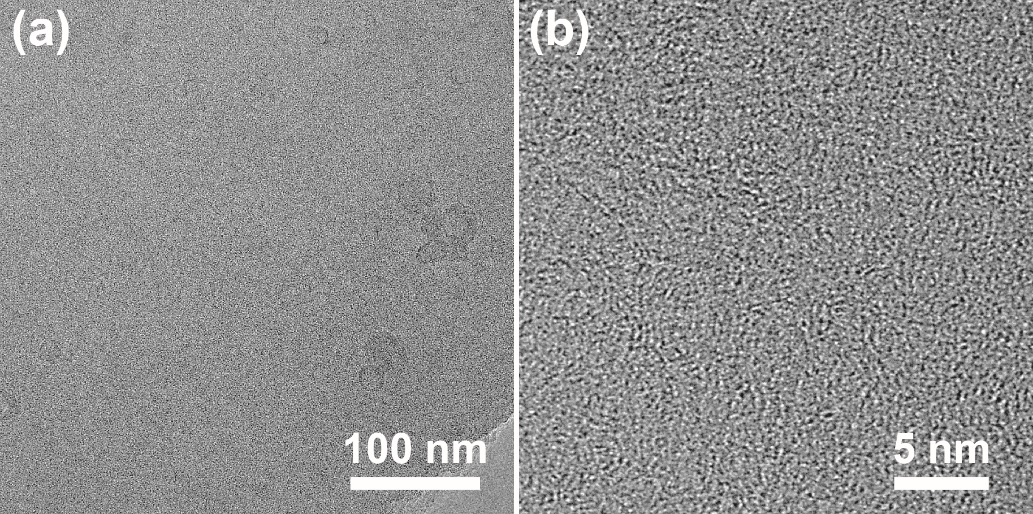


**Supplementary Figure 2.** (a) TEM image and (b) HRTEM image of nanoporous carbon substrate.


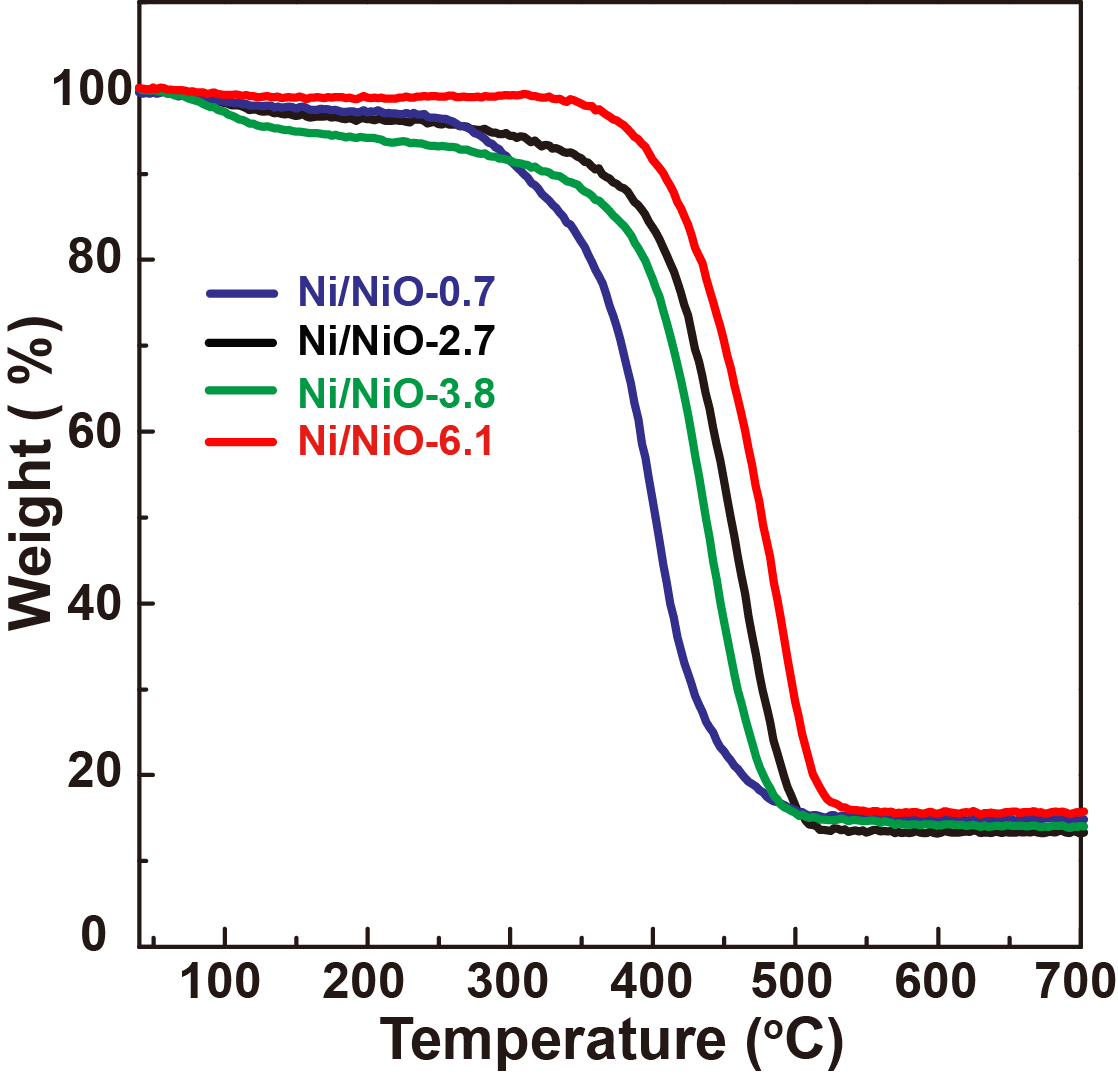


**Supplementary Figure 3.** TG curves of Ni/NiO-0.7, -2.7, -3.8, and -6.1 measured in air.

As shown in TG curves, the losses of initial weight below 100 °C should be ascribed to the evaporation of adsorbed species. The sharp weight loss should be from the combustion of carbon. The remaining weights after heating to 600 ^o^C are 13.37, 11.97, 11.07, and 12.35 wt% of Ni/NiO-0.7, -2.7, -3.8, and -6.1, respectively. Accordingly, the Ni contents are calculated as follows:

For Ni/NiO- 0.7: m% (Ni) = residual mass ⨉ M (Ni) / M (NiO)

=13.37 wt% ⨉ 58.69/74.69 ≈ 10.51 wt%

For Ni/NiO-2.7: m% (Ni) = residual mass ⨉ M (Ni) / M (NiO)

=15.22 wt% ⨉ 58.69/74.69 ≈ 11.96 wt%

For Ni/NiO-3.8: m% (Ni) = residual mass ⨉ M (Ni) / M (NiO)

=14.10 wt% ⨉ 58.69/74.69 ≈ 11.07 wt%

For Ni/NiO-6.1: m% (Ni) = residual mass ⨉ M (Ni) / M (NiO)

=15.72 wt% ⨉ 58.69/74.69 ≈ 12.35 wt%


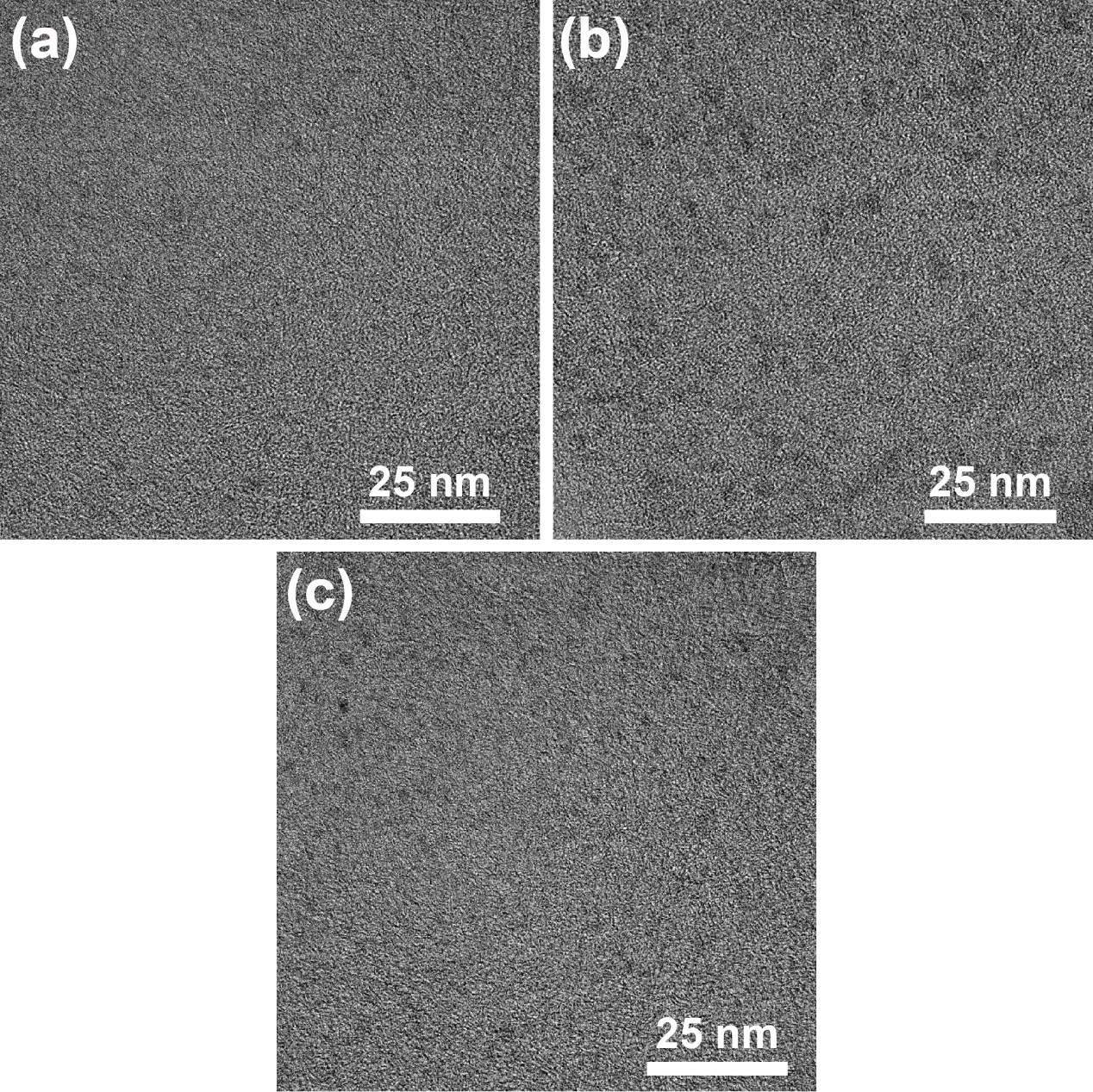


**Supplementary Figure 4.** TEM images of Ni/NiO-0.7.


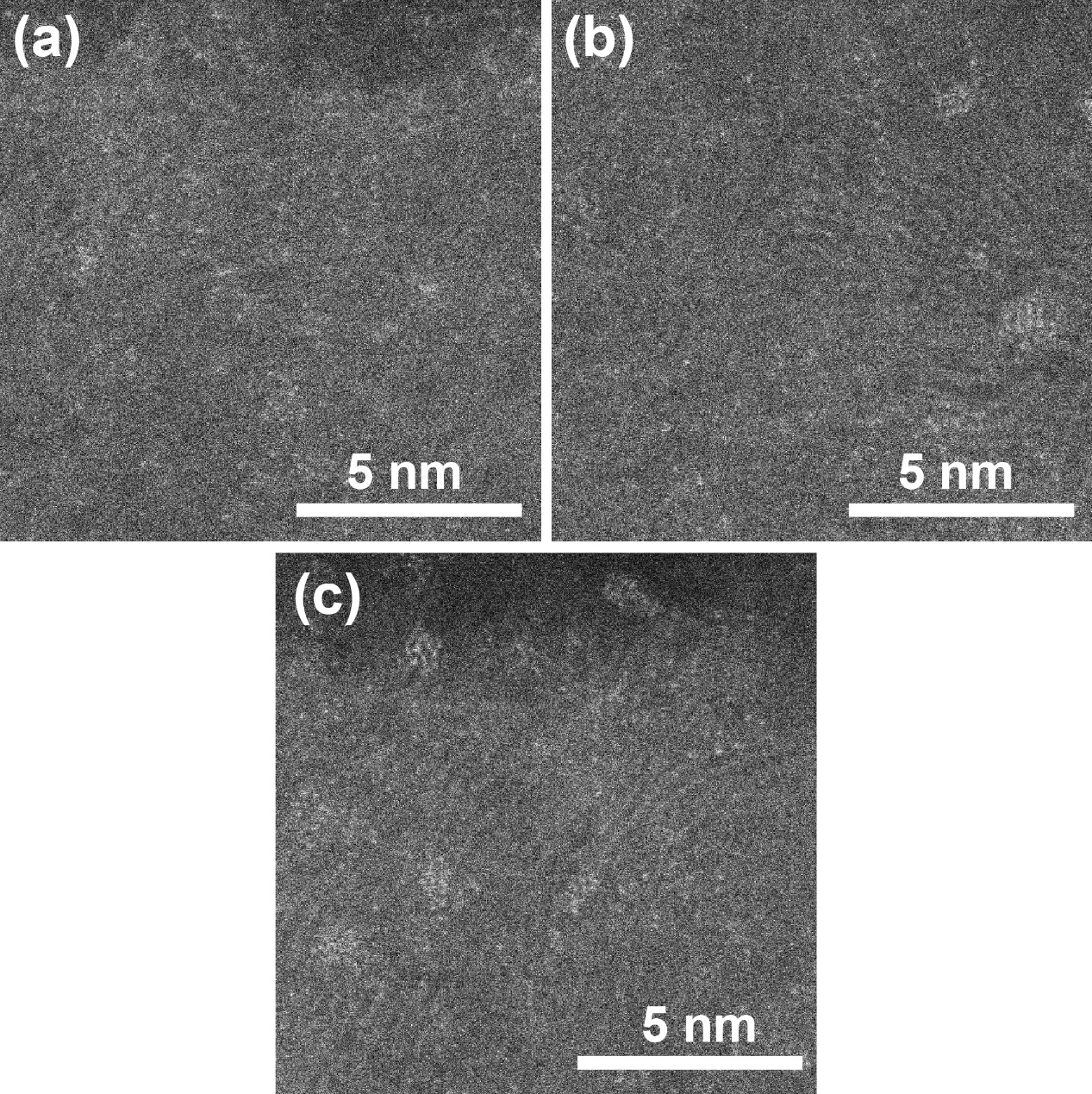


**Supplementary Figure 5.** HAADF-STEM images of Ni/NiO-0.7.


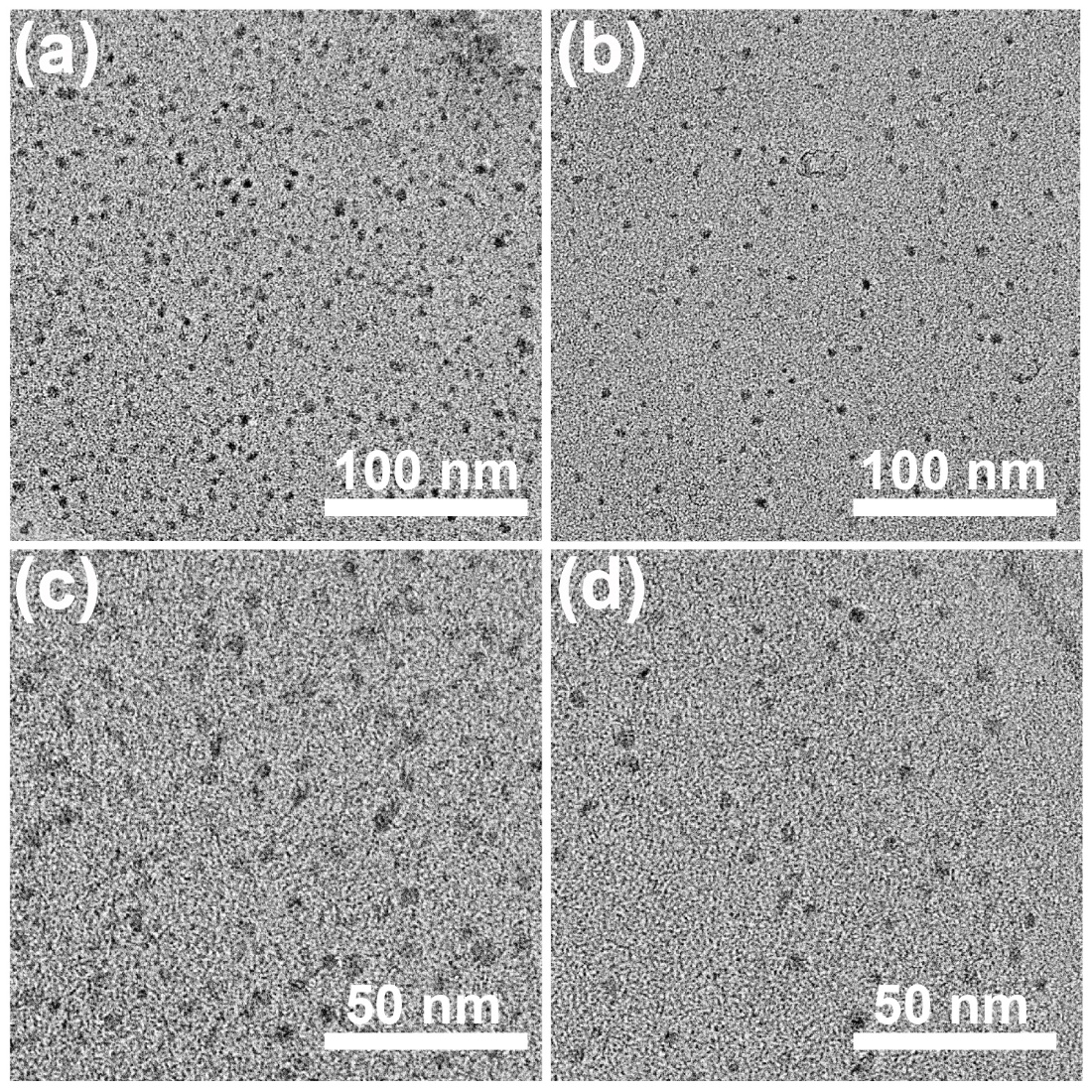


**Supplementary Figure 6.** TEM images of Ni/NiO-2.7.


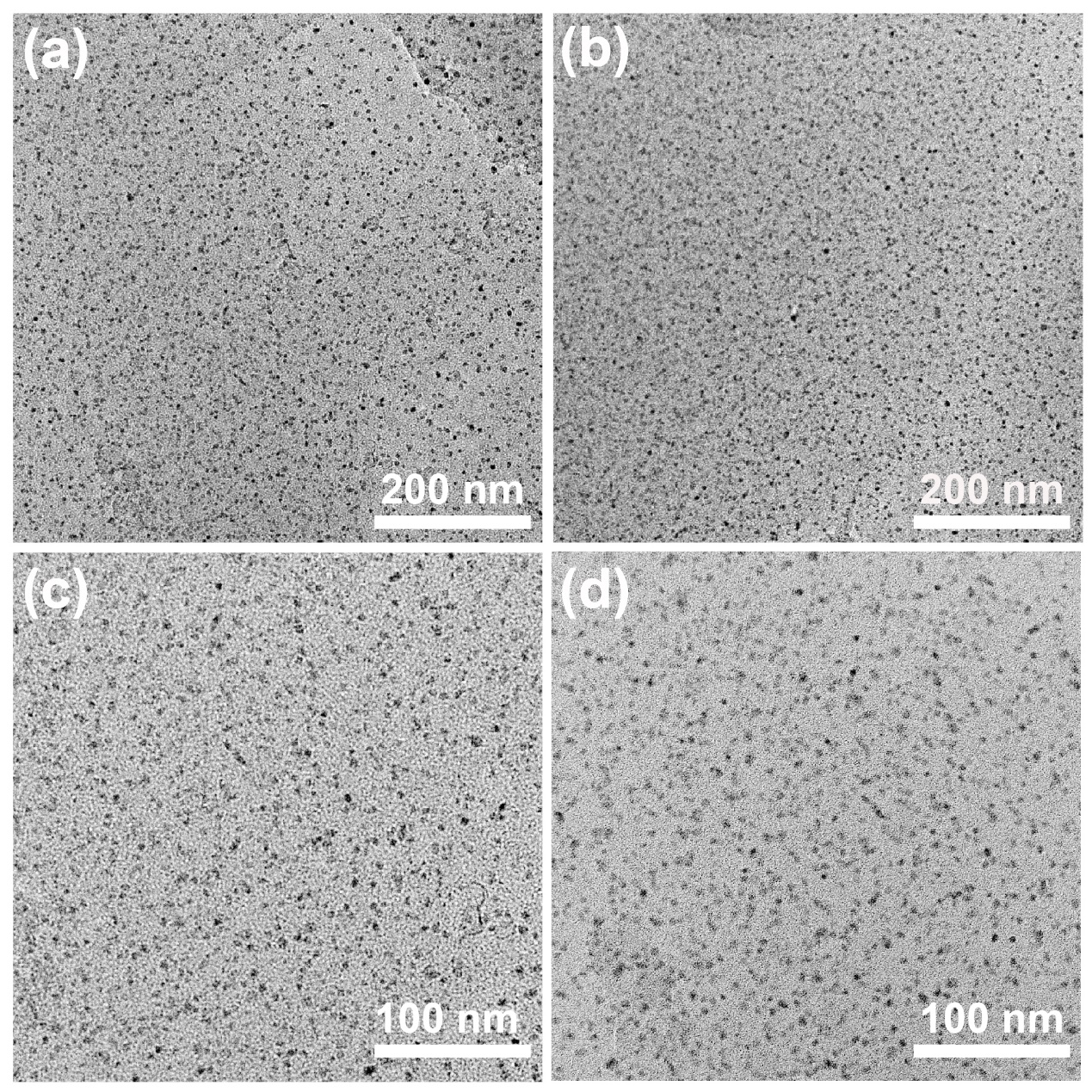


**Supplementary Figure 7.** TEM images of Ni/NiO-3.8.


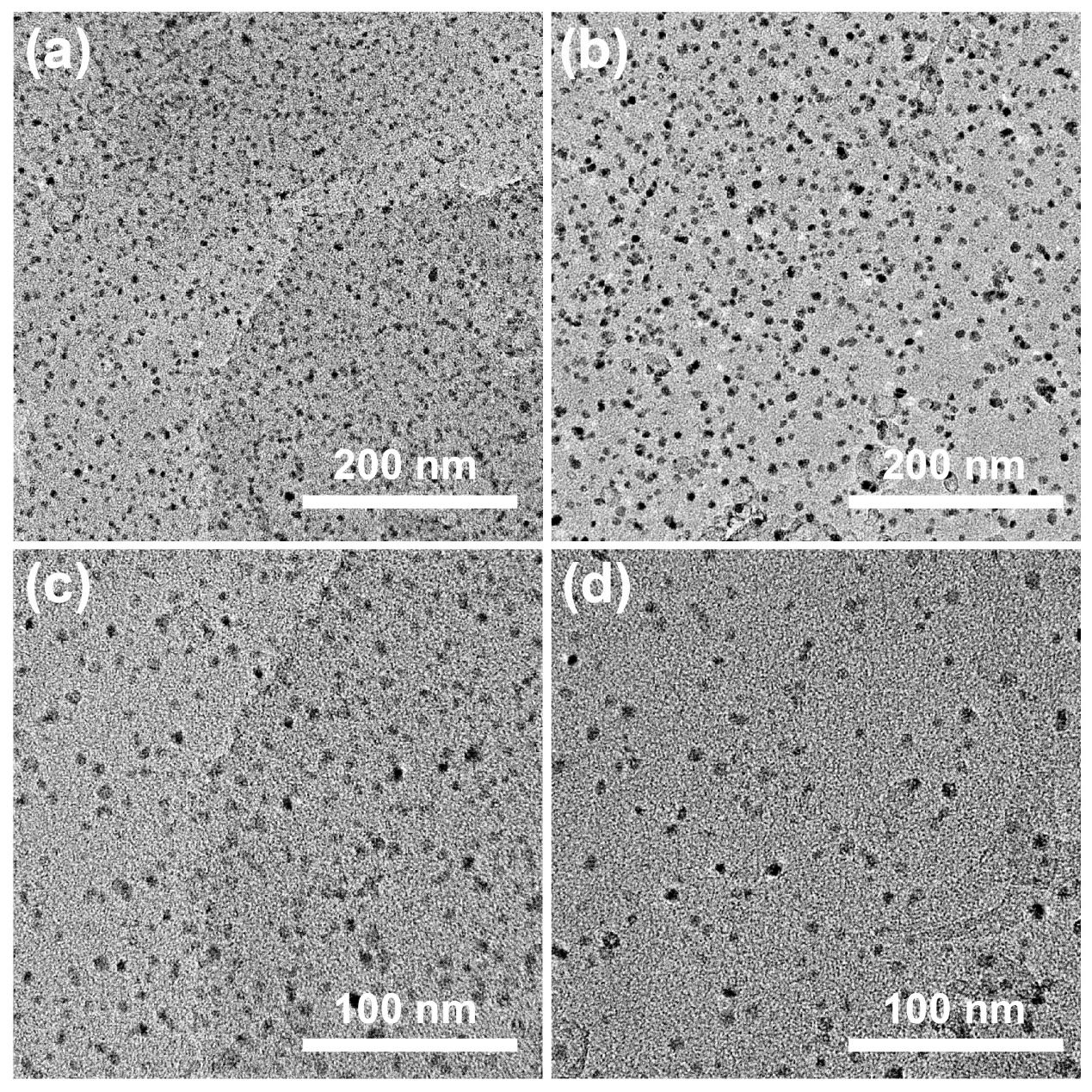


**Supplementary Figure 8.** TEM images of Ni/NiO-6.1.


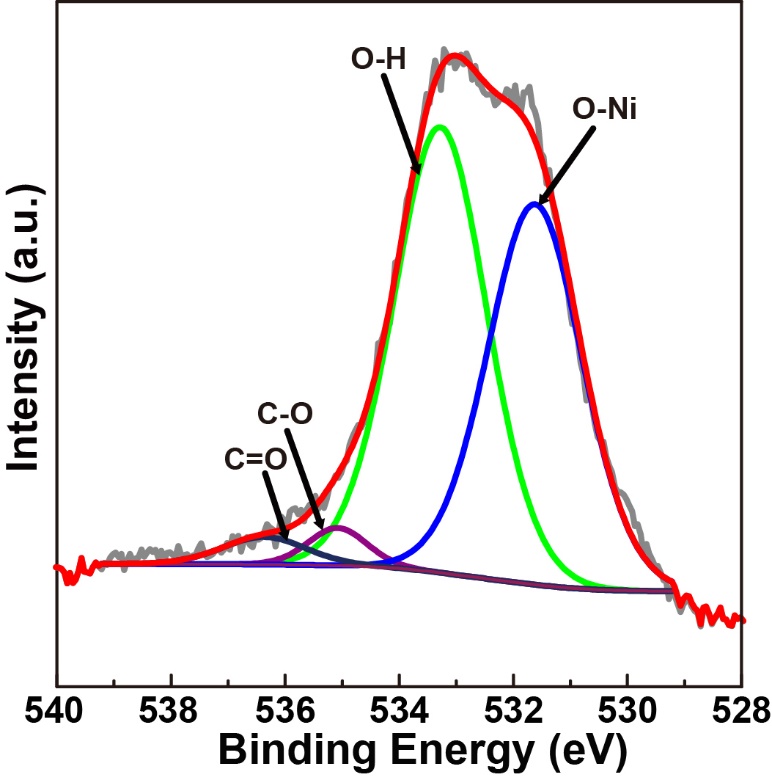


**Supplementary Figure 9.** High-resolution O 1s XPS spectrum of Ni/NiO-3.8.


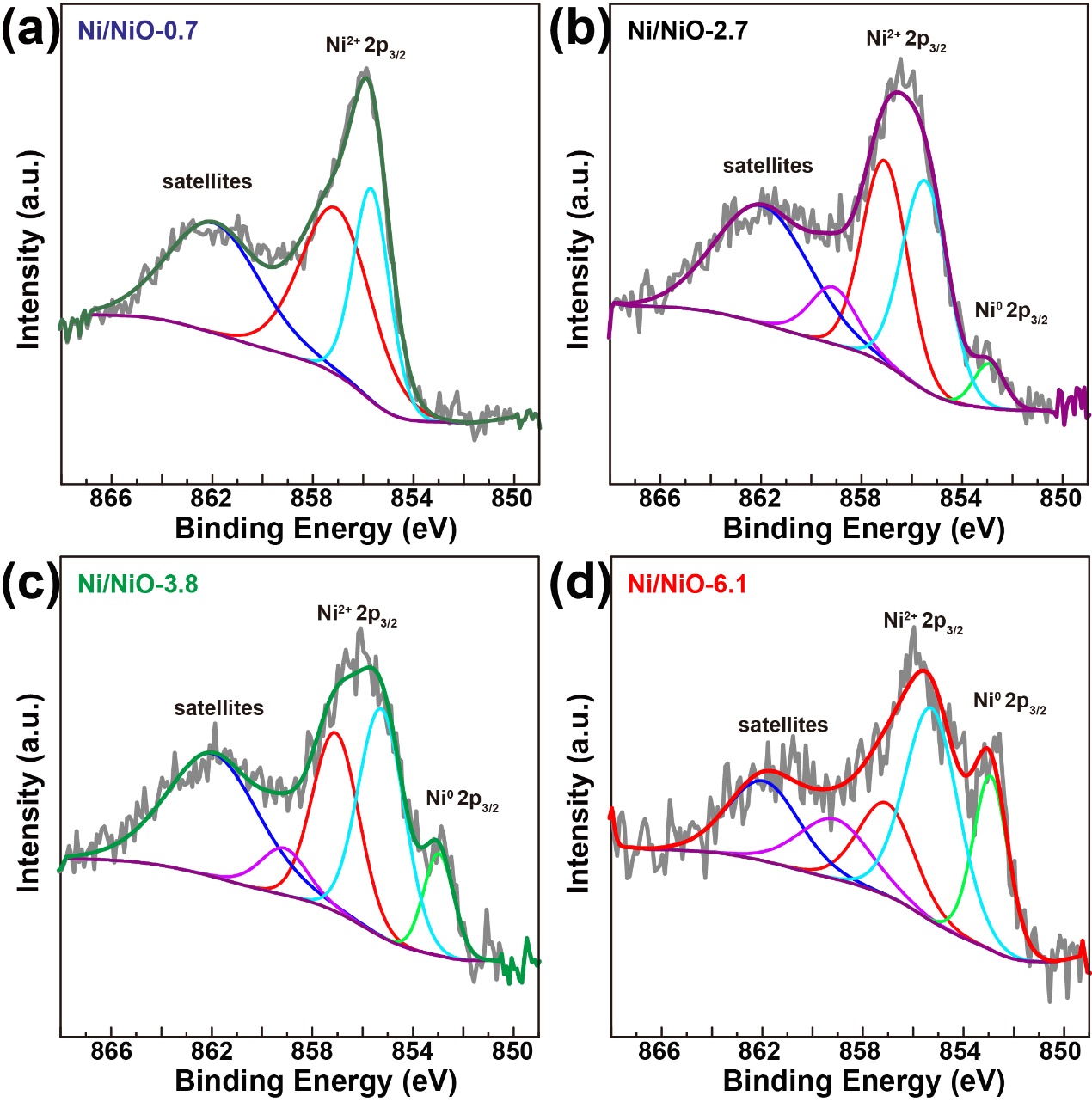


**Supplementary Figure 10.** High-resolution Ni 2p_3/2_ XPS spectra of (a) Ni/NiO-0.7, (b) -2.7, (c) -3.8 and (d) -6.1.


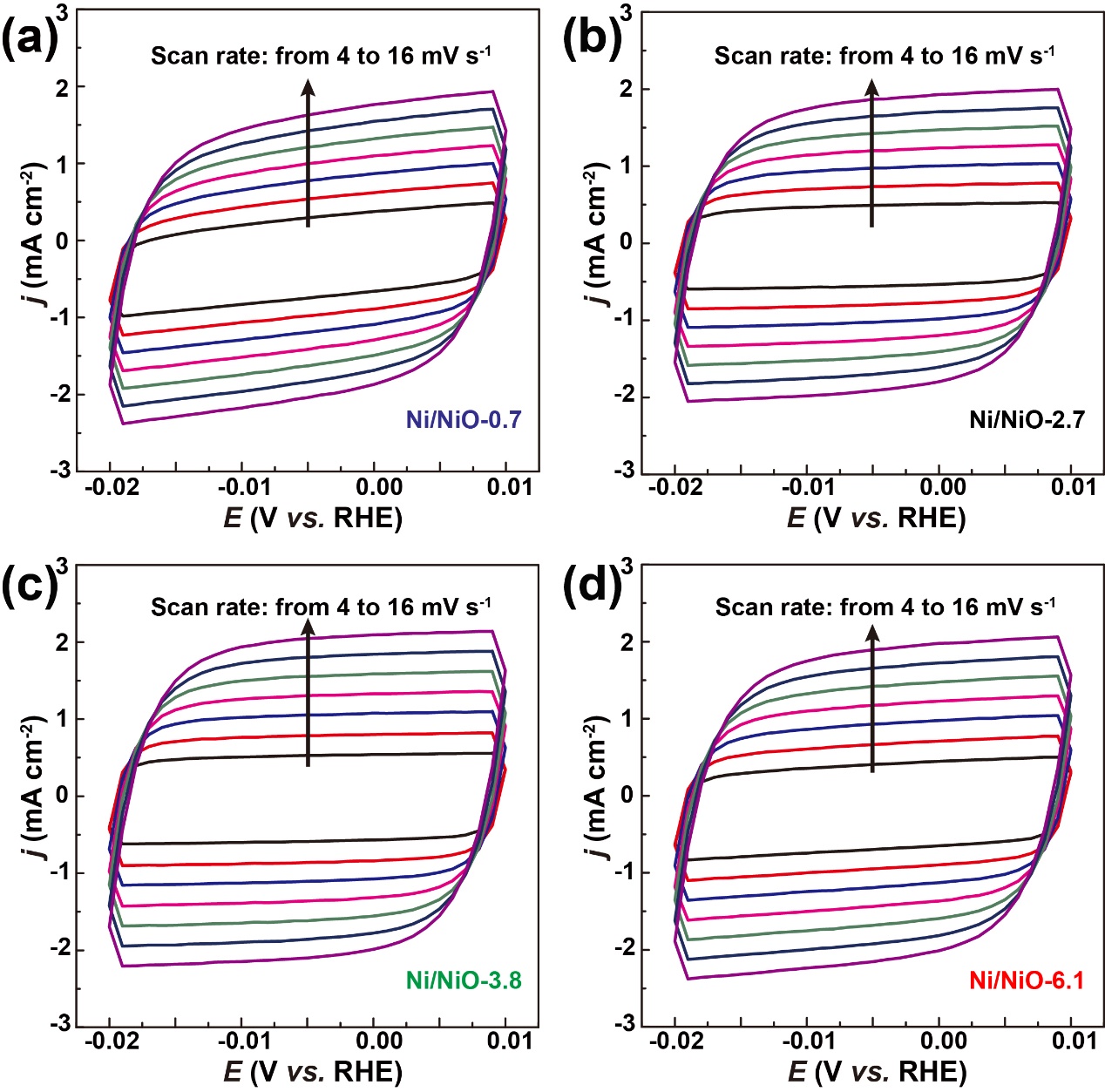


**Supplementary Figure 11.** Cyclic voltammetric curves of (a) Ni/NiO-0.7, (b) -2.7, (c) -3.8, and (d) -6.1, recorded at different scan rates within the range of -0.02 to + 0.01 V (*vs.* RHE) where no faradaic reactions occurred.


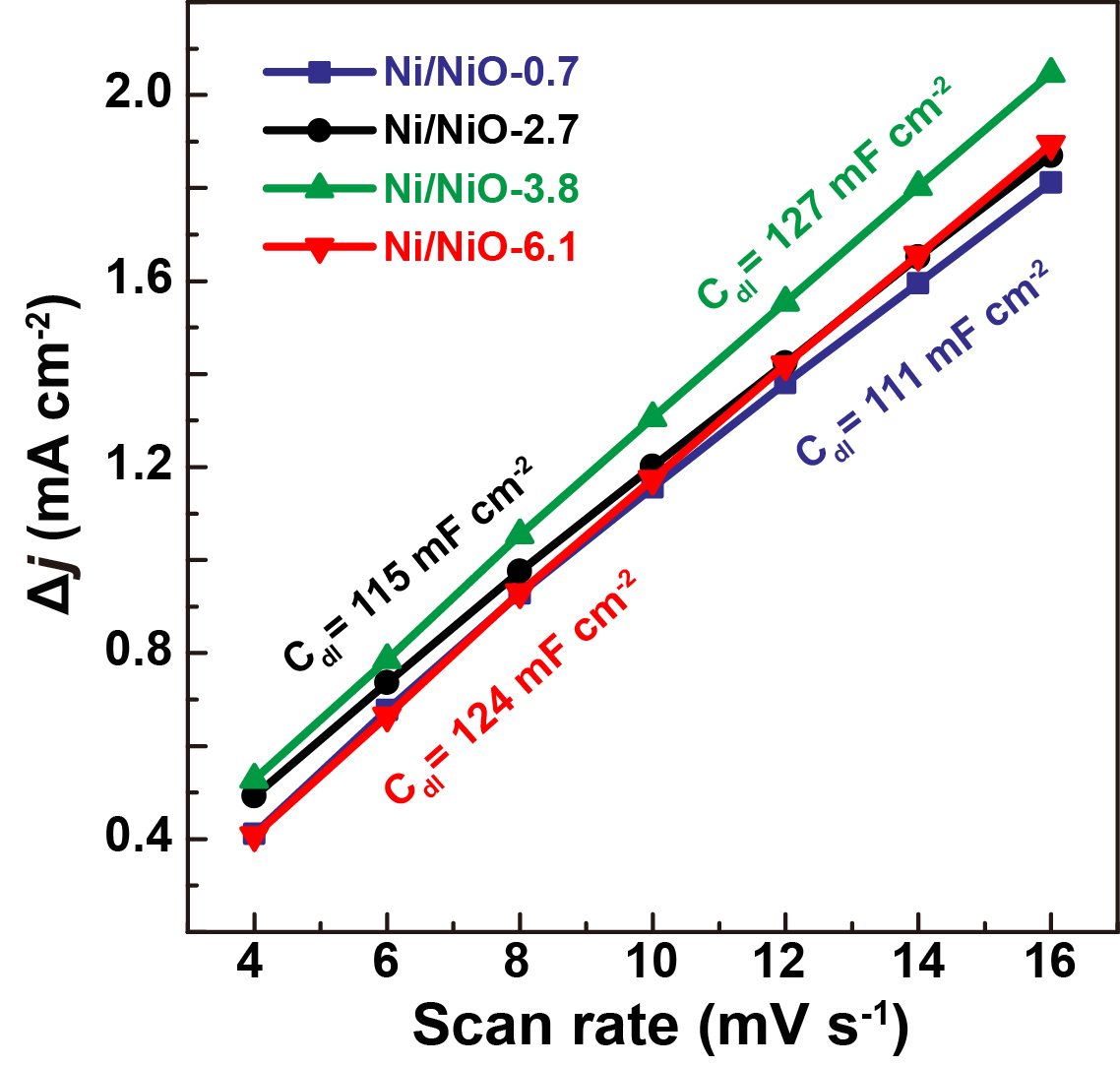


**Supplementary Figure 12.** The capacitive current determined by plotting current density at the potential of -5 mV as a function of scan rates for Ni/NiO samples.


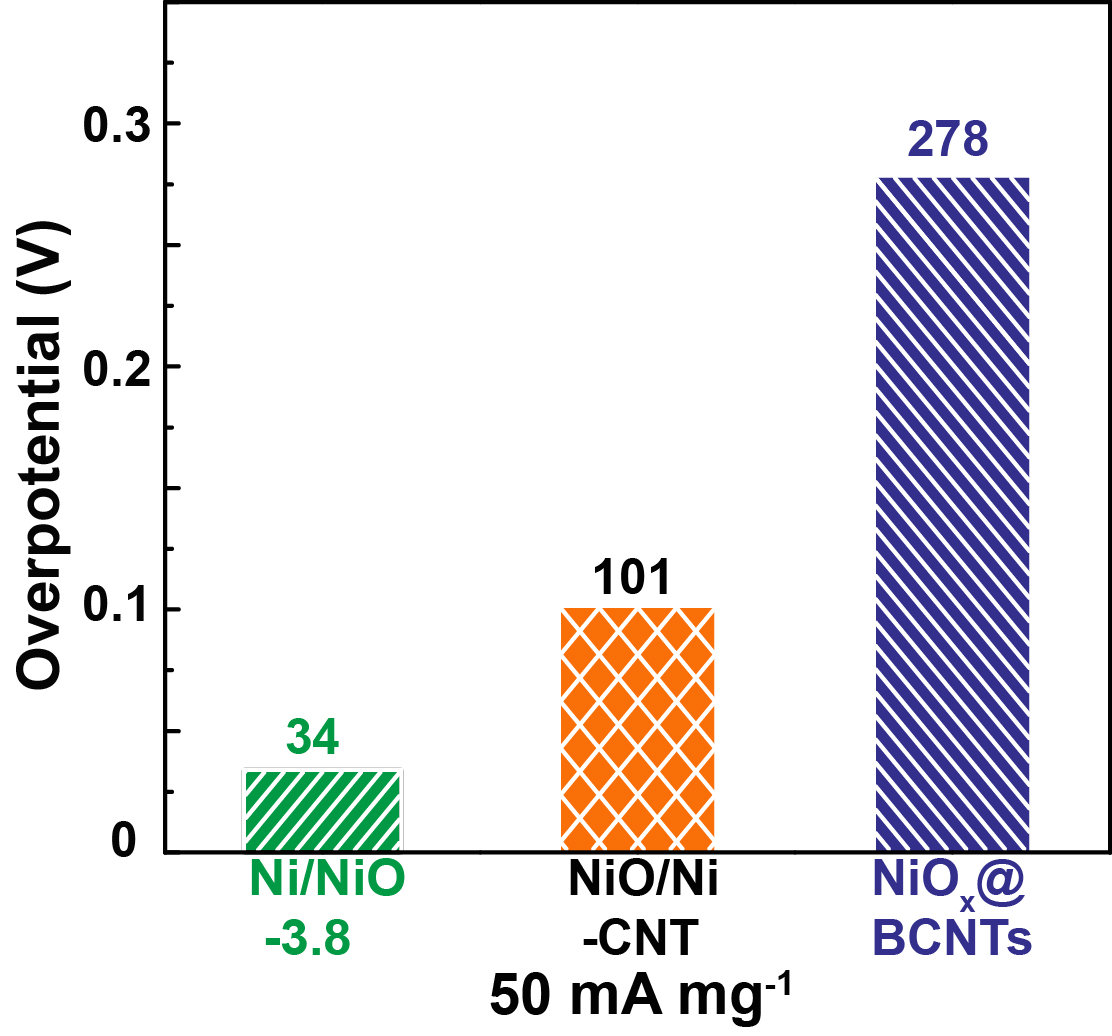


**Supplementary Figure 13.** Comparison of overpotentials at 50 mA mg^-1^ for the state-of-the-art Ni/NiO HER catalysts (Ni/NiO-3.8 in this work, NiO/Ni-CNT [2] and NiO_x_@BCNTs [3]) in 1 M KOH.


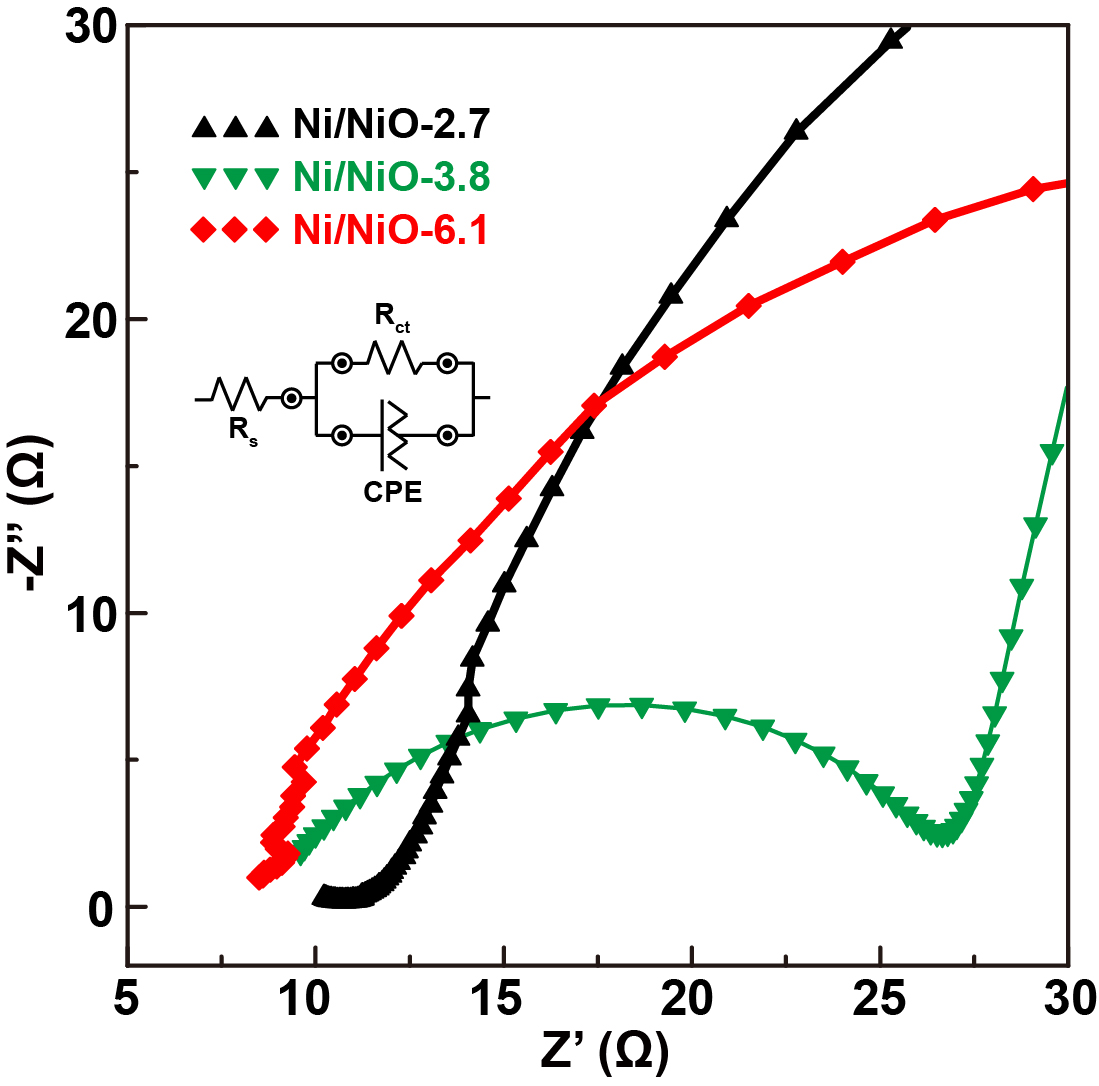


**Supplementary Figure 14.** EIS Nyquist plots at an overpotential of 0.226 V for Ni/NiO samples.

**
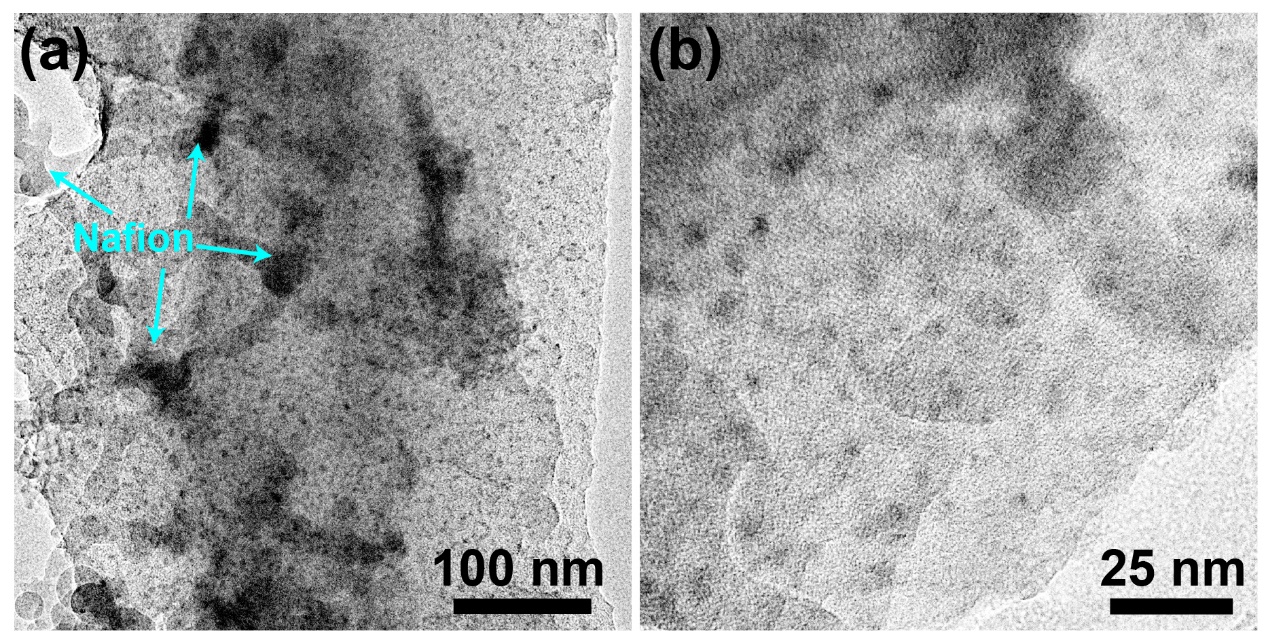
**

**Supplementary Figure 15.** TEM images of Ni/NiO-3.8 after stability tests. The morphology and size of Ni/NiO nanocrystals show no appreciable change.

**
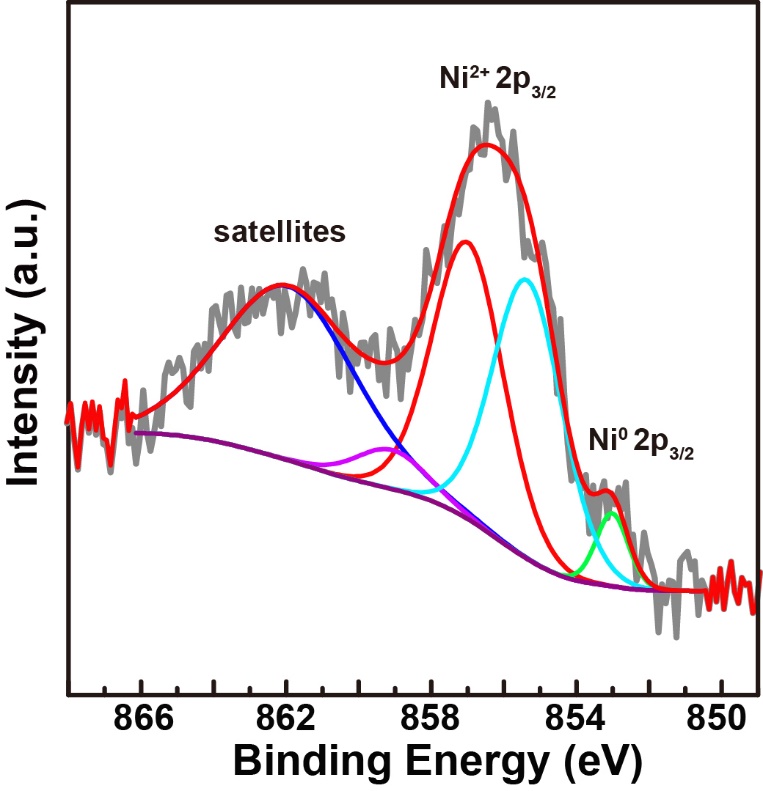
**

**Supplementary Figure 16.** High-resolution Ni 2p_3/2_ XPS spectrum of Ni/NiO-3.8@100 ^o^C.


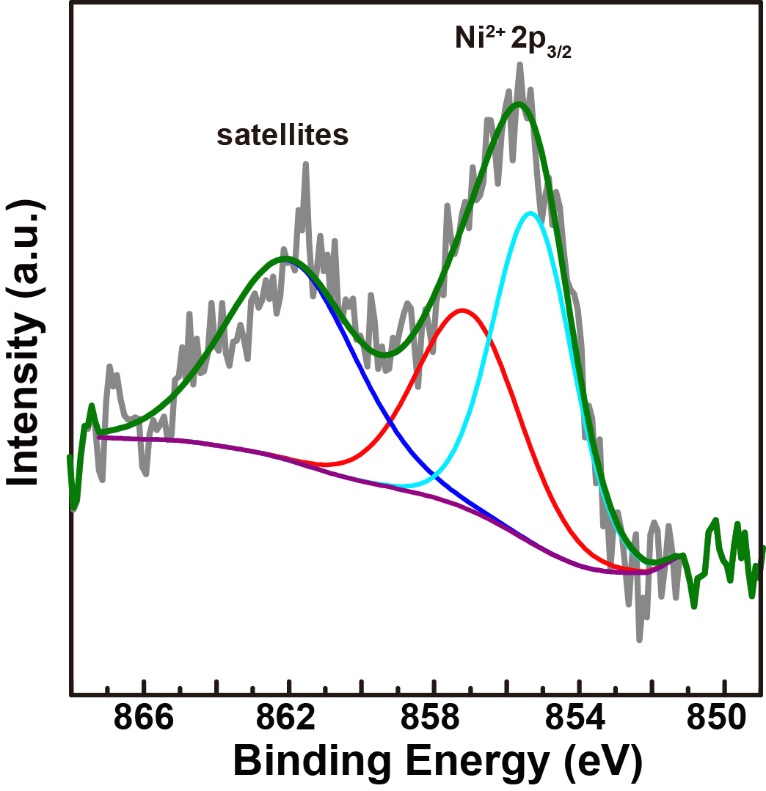


**Supplementary Figure 17.** High-resolution Ni 2p_3/2_ XPS spectrum of Ni/NiO-3.8@200 ^o^C.


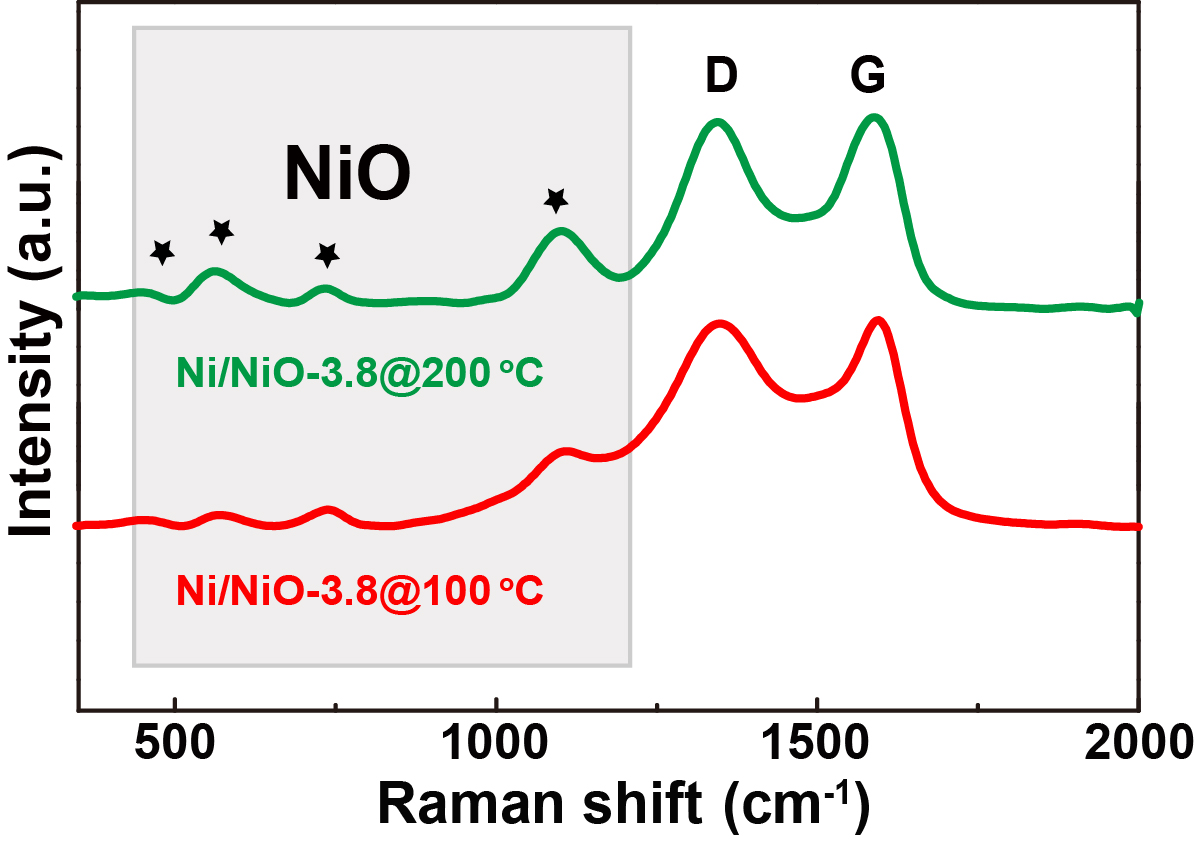


**Supplementary Figure 18.** Raman spectra of Ni/NiO-3.8@100 ^o^C and -@200 ^o^C. The shaded region highlights the Raman vibrations of NiO.


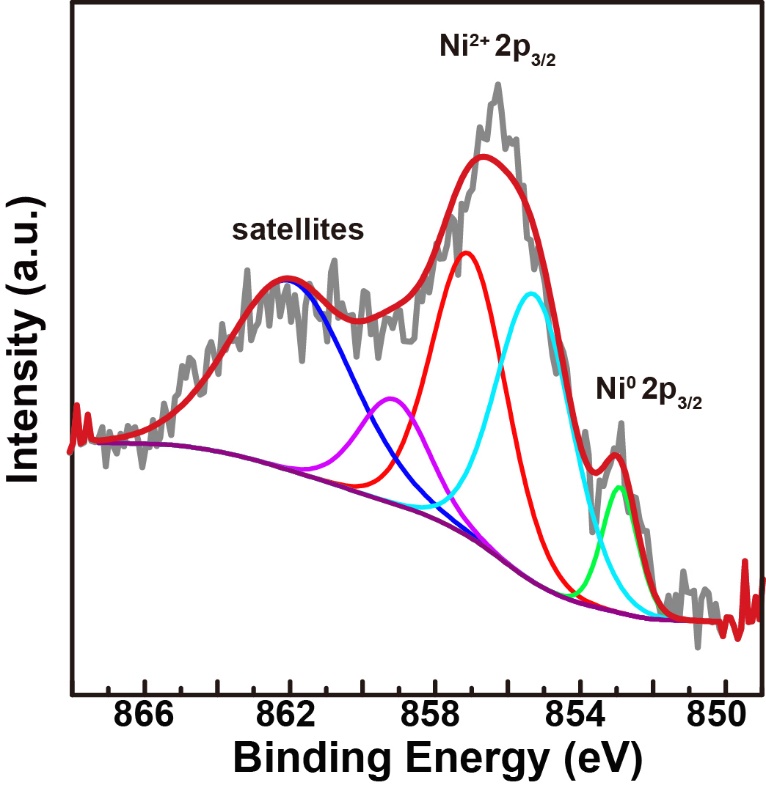


**Supplementary Figure 19.** High-resolution Ni 2p_3/2_ XPS spectrum of Ni/NiO-6.1@100 ^o^C.


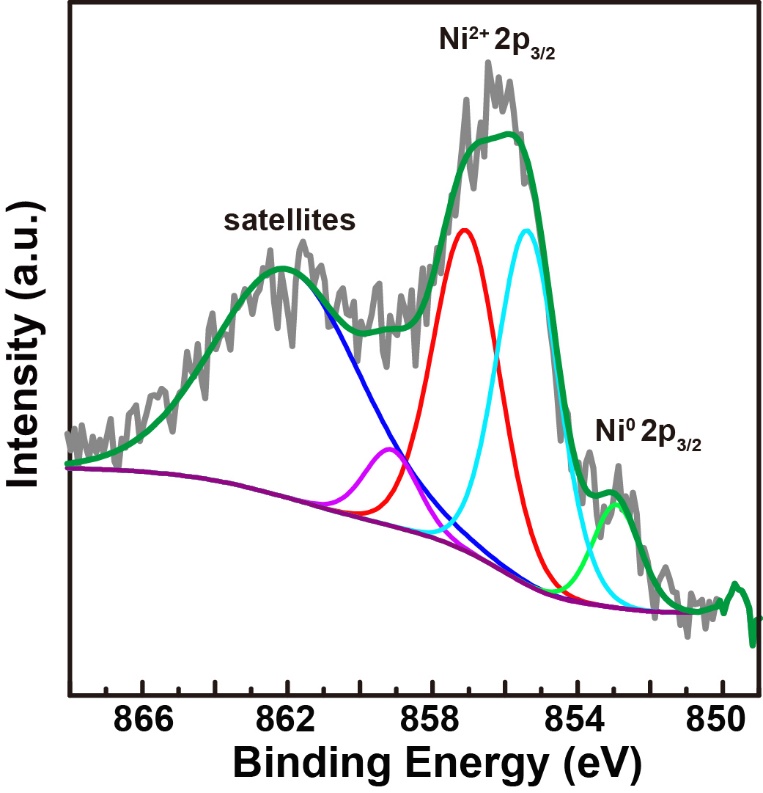


**Supplementary Figure 20.** High-resolution Ni 2p_3/2_ XPS spectrum of the Ni/NiO-6.1@150 ^o^C.


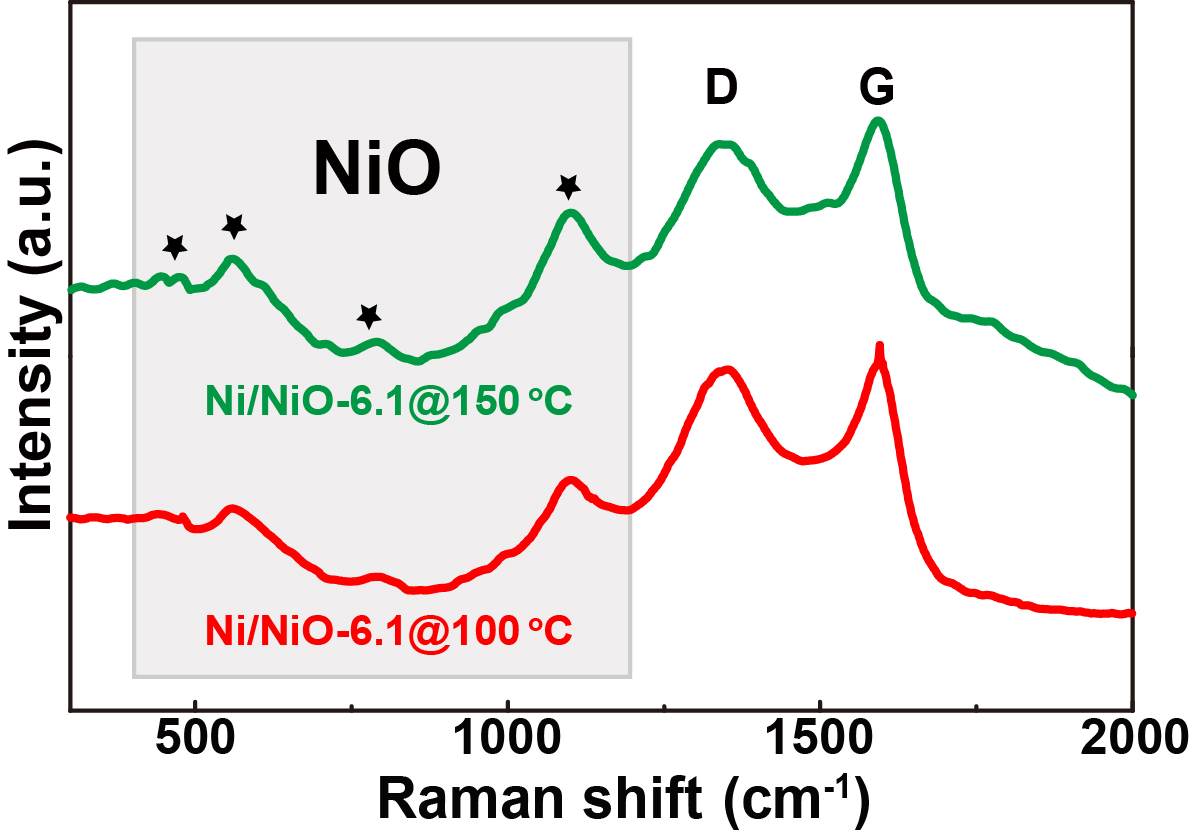


**Supplementary Figure 21.** Raman spectra of Ni/NiO-6.1@100 ^o^C and -@150 ^o^C. The shaded region highlights the Raman vibrations of NiO.

**Supplementary Table 1.** Ni/NiO ratios calculated from XPS fittings of all samples.

| Samples | Ni/NiO-0.7 | Ni/NiO-2.7 | Ni/NiO-3.8 | Ni/NiO-6.1 | Ni/NiO-3.8@100 ^o^C | Ni/NiO-3.8@200 ^o^C | Ni/NiO-6.1@100 ^o^C | Ni/NiO-6.1@150 ^o^C |
| --- | --- | --- | --- | --- | --- | --- | --- | --- |
| Ni/NiO (%) | ~0 | 13.2 | 23.7 | 59.5 | 11.5 | ~0 | 27.8 | 15.8 |

**Supplementary Table 2.** HER performance comparisons for recently reported state-of-the-art Ni-based catalysts in 1 M KOH.

| Catalyst | η@10 mA  cm^-2^ [mV] | Tafel slope  [mV dec^-1^] | Mass loading  [mg cm^-2^] | Reference |
| --- | --- | --- | --- | --- |
| **Ni/NiO-3.8** | **90** | **41** | **0.09**  **(active mass)** | **This Work** |
| Ni NP\|Ni-N-C | 147 | 114 | 0.24 | *Energy Environ. Sci.*  **2019,** *12*, 149-156 |
| Ni_2_P/Fe_2_P | 121 | 67 | 0.27 | *Adv. Energy Mater.*  **2018,** *8*, 1800484 |
| NiFe-MOF  (0.1 M KOH) | 134 | -- | 0.3 | *Nat. Commun.*  **2017,** *8*, 15341 |
| NiS_2_/MoS_2_ HNW | 204 | 65 | 0.2 | *ACS Catal.*  **2017,** *7*, 6179-6187 |
| MoS_2_/Ni_2_S_3_ | 110 | 83 | 9.7 | *Angew. Chem., Int. Ed.*  **2016,** *55*, 6702-6707 |
| Ni/NiS | 230 | 123.3 | 11.04 | *Adv. Funct. Mater.*  **2016,** *26*, 3314-3323 |
| Ni/NiP | 130 | 58.5 | 10.58 |  |
| NiSe/NF | 96 | 120 | 2.8 | *Angew. Chem., Int. Ed.*  **2015,** *54*, 9351-9355 |
| Ni_5_P_4_ on Nickel foil | 150 | 53 | -- | *Angew. Chem., Int. Ed.*  **2015,** *54*, 12361-12365 |
| Ni-NiO/N-rGO | 260 | 67 | 0.21 | *Adv. Funct. Mater.*  **2015,** *25*, 5799-5808 |
| NiO/Ni-CNT | ＜100 | 82 | 0.28  (active mass) | *Nat. Commun.*  **2014,** *5*, 4695 |
| Ni_2_P | 210 | -- | 1 | *J. Am. Chem. Soc.*  **2013,** *135*, 9267-9270 |
| Ni wire  （1M NaOH） | 350 | -- | -- | *ACS Catal.*  **2013,** *3*, 166-169 |
| Ni-BDT-A  (carbon cloth) | 80 | 70 | 0.3 | *Chem*  **2017**, *3*, 122–133 |
| NiS_2_-A  (Ni foam) | 67 | 72 | 1.6 | *Nano Energy*  **2017**, *41*, 148–153 |
| Ni-NiO/C HPPAs  (Ni foam) | 49.48 | 74 | 0.51 | *ACS. Appl. Mater. Interfaces*  **2018,** *10*, 38906-38914 |
| Ni-NiO/C HPPs  (Ni foam) | 94.98 | 84 |  |  |
| Ni/NiO-400  (carbon cloth) | 41 | 59 | 1 | *Applied Catalysis B: Environmental*  **2019,** *244*, 732–739 |

**Supplementary Reference**

1. Kibsgaard J, Jaramillo T F and Besenbacher F. Building an Appropriate Active-Site Motif into a Hydrogen-Evolution Catalyst with Thiomolybdate [Mo_3_S_13_]^2−^ Clusters. *Nat. Chem.* 2014; **6**: 248-253.

2. Gong M, Zhou W and Tsai M-C *et al.* Nanoscale Nickel Oxide/Nickel Heterostructures for Active Hydrogen Evolution Electrocatalysis. *Nat. Commun.* 2014; **5**: 4695.

3. Wang J, Mao S and Liu Z *et al.* Dominating Role of Ni^0^ on the Interface of Ni/NiO for Enhanced Hydrogen Evolution Reaction. *ACS Appl. Mater. Interfaces* 2017; **9**: 7139-7147.
